# The Pharmacokinetics, Tissue Distribution, Metabolism, and Excretion of Pinostrobin in Rats: Ultra-High-Performance Liquid Chromatography Coupled With Linear Trap Quadrupole Orbitrap Mass Spectrometry Studies

**DOI:** 10.3389/fphar.2020.574638

**Published:** 2020-11-26

**Authors:** Xiaoya Sun, Xiaojun Liu, Suiqing Chen

**Affiliations:** ^1^ School of Pharmacy, Henan University of Chinese Medicine, Zhengzhou, China; ^2^ Collaborative Innovation Center for Respiratory Disease Diagnosis and Treatment & Chinese Medicine Development of Henan Province, Henan University of Chinese Medicine, Zhengzhou, China

**Keywords:** pinostrobin, UPLC-LTQ orbitrap-MS/MS, pharmacokinetics, tissue distribution, metabolites, excretion

## Abstract

Pinostrobin is a natural flavonoid found in various plants, well known for its wide range of pharmacological activities. However, there are few reports regarding the pharmacokinetics, tissue distribution, metabolism, and excretion of pinostrobin in rats after oral administration as a single compound. Therefore, we established a method using ultra-high-performance liquid chromatography coupled with linear trap quadrupole orbitrap mass spectrometry (UPLC-LTQ orbitrap-MS/MS) to determine pinostrobin and its metabolites in rat plasma, urine, feces, bile, and tissue homogenates. Pharmacokinetic parameters were measured. The large apparent volume of distribution implied that pinostrobin preferentially bound to tissues and preferably remained within the body. Based on previous pharmacological studies of its antiulcer, anti-*HP*, anti-inflammatory, and antioxidant activities, pinostrobin is mostly distributed in the gastrointestinal tract, indicating its potential as an effective component of traditional Chinese medicines for the treatment of peptic ulcers. Furthermore, 30 flavonoid metabolites were screened using UPLC-LTQ orbitrap-MS/MS. The metabolism pathways (mainly hydroxylation, demethylation, glucuronidation, and sulfation) of pinostrobin in rats have also been proposed. A small amount of pinostrobin in its parent form is excreted through the urine, feces, and bile, indicating that it is mainly metabolized *in vivo*. In this study, we systemically investigated the pharmacokinetics, tissue distribution, metabolism, and excretion of pinostrobin in rats. Our results provide a significant basis for the clinical development and application of pinostrobin as well as traditional Chinese medicines containing pinostrobin.

## Introduction

Peptic ulcers are a common disease encountered in the clinic. Epidemiology estimated the morbidity rate of peptic ulcer and its related disorders to be as high as 10%, especially in the past 10 years, and its incidence continues to increase (Lanas and Chan, 2017; Kuna et al., 2019). For the past few years, *Lindera reflexa* Hemsl., *Boesenbergia rotunda* (L.) Mansfield, *Dysphania graveolens*, and *Teloxys graveolens* have been used to treat gastrointestinal disorders, including peptic ulcers. Flavonoids, especially pinostrobin, are the main active components of these plants that contribute to the effective treatment of peptic ulcer (Meckes et al., 1998; Sun et al., 2016; Déciga-Campos et al., 2017; Kanchanapiboon et al., 2020).

Pinostrobin, a widely studied dietary bioflavonoid, was discovered in the heartwood of pine (*Pinus strobus* L.) more than 7 decades ago (Erdtman, 1944). In addition to Pinaceae, pinostrobin has also been found in more than 10 families such as Lauraceae, Zingiberaceae, Fabaceae, and Polygonaceae (Patel and Bhutani, 2014; Chen et al., 2015; Gómez-Betancur et al., 2015; Dzoyem et al., 2017; Vasas et al., 2020). Pinostrobin content has been found to control the quality of Linderae Reflexae Radix. It is found more abundantly in roots than other secondary metabolites, such as pinocembrin and pinosylvin (Wang et al., 2016). Pinostrobin is known to have various pharmacological activities (Patel et al., 2016), including antiulcer, anti-*Helicobacter pylori* (Bhamarapravati et al., 2006), antioxidant, anti-inflammatory, anticancer (Jaudan et al., 2018; Jones and Gehler, 2020; Sopanaporn et al., 2020), antidiarrheal, antiviral (Wu et al., 2011), antimicrobial (Hernández Tasco et al., 2020), anti-Alzheimer’s, antiprotozoal, antinociceptive (Déciga-Campos et al., 2017), antimutagenic, antiplatelet (Zhang et al., 2017), antiproliferative (Siekmann et al., 2013; Jadaun et al., 2017), antileukemic (Smolarz et al., 2005), antiosteoporotic (Gu et al., 2017), and antiparasitic properties (Vechi et al., 2020). Moreover, pinostrobin can protect the gastric mucosa by reducing the ulcer area and mucosal content and reducing or eliminating submucosal edema and leukocyte infiltration. Pinostrobin has a significant, dose-dependent protective effect on ethanol-induced gastric mucosal injury by scavenging free radicals produced by ethanol through the activation of cellular antioxidant defenses (Abdelwahab et al., 2011). Meanwhile, it exhibits nontoxic and nongenotoxic effects (Charoensin et al., 2010). However, to the best of our knowledge, the pharmacokinetic processes of pinostrobin have not yet been explained clearly.

Pharmacokinetic profiling, including absorption, distribution, metabolism, and excretion (ADME) processes, is vital to understanding the *in vivo* behavior and mechanism of action of compounds (Zeng et al., 2019). To date, there have been only a few studies regarding the determination of pinostrobin in rat plasma using high-performance liquid chromatography-ultraviolet (HPLC-UV) or HPLC-MS/MS after intravenous and intragastric administration, and only some of the pharmacokinetic parameters of pinostrobin have been reported (Hua et al., 2011; Sayre et al., 2013; Sayre et al., 2015). Hence, it is meaningful to explore the ADME processes of pinostrobin in rats after oral administration as a single compound.

In this study, we systemically investigated the pharmacokinetics and tissue distribution, metabolism, and excretion properties of pinostrobin in rats after a single oral administration. The pharmacokinetic parameters were consistent with those reported in previous studies. Pinostrobin was mostly distributed in the gastrointestinal tract. The amount of pinostrobin excreted via urine, feces, and bile in the parent form was less than 1.567%. Thirty flavonoid metabolites were identified or partially identified in biosamples collected after dosing. In addition, we proposed metabolic pathways in rats. This study provides helpful information for the clinical study of pinostrobin and traditional Chinese medicines containing pinostrobin.

## Materials and Methods

### Chemicals and Materials

**FIGURE 1:**
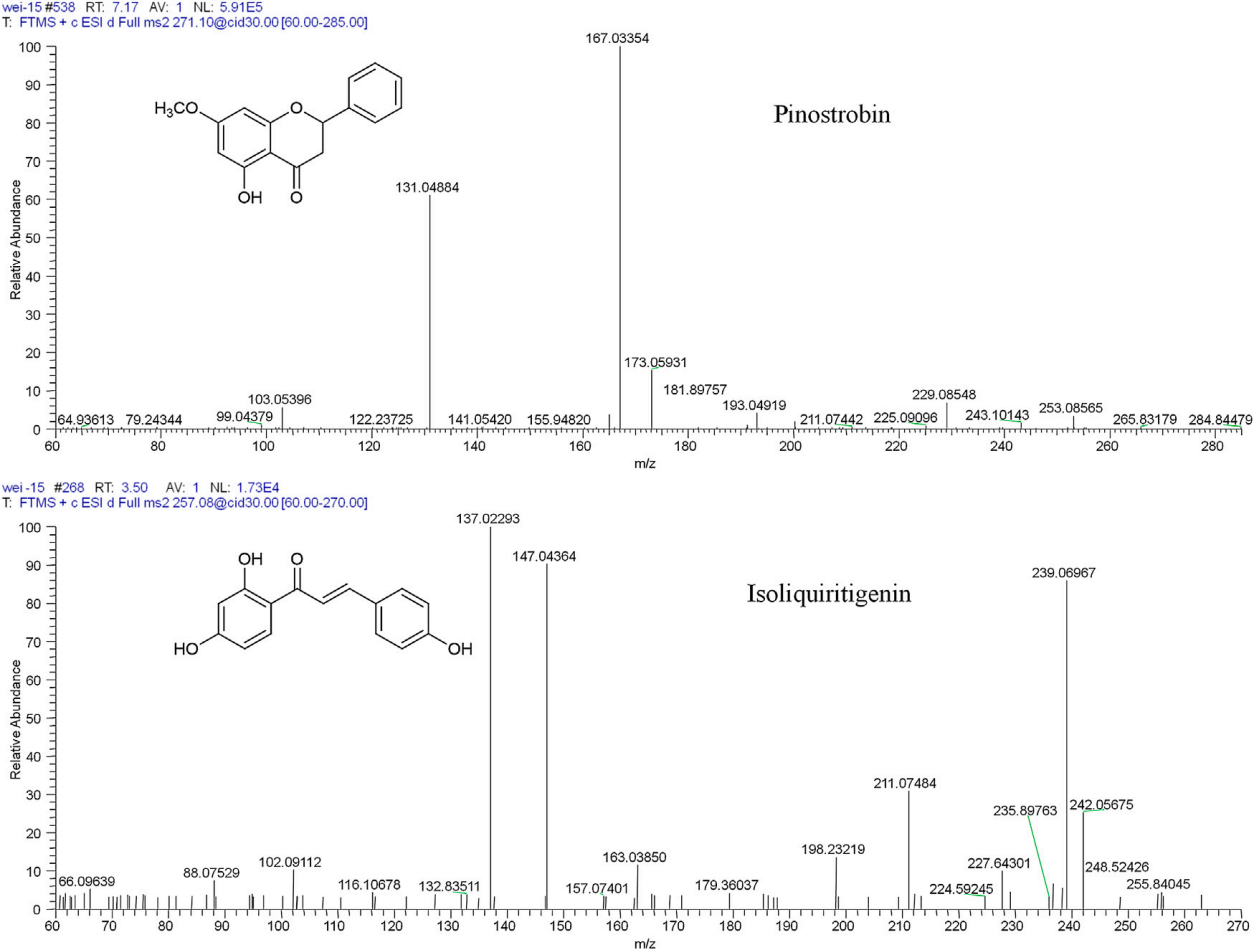
The chemical structures and mass spectra of pinostrobin and isoliquiritigenin (IS).

Pinostrobin was isolated from the roots of *L. reflexa* Hemsl. in our laboratory. Its structure (Figure 1) was unequivocally elucidated using spectroscopic methods (IR, MS, ^1^H-NMR, and ^13^C-NMR) (Chen et al., 2015; Sun et al., 2016). The absolute stereochemistry of pinostrobin was found to be 2S by comparing the ECD spectra (Supplementary Figure S1) with previously published data (Gaffield, 1970; Su et al., 2003). Its purity was determined to be above 98% by normalizing the peak area using an HPLC-diode array detector. Isoliquiritigenin (HPLC ≥ 98%) obtained from Shanghai Yuanye Bio-Technology Co., Ltd., was used as the internal standard (IS). Ultrapure water was prepared using a Milli-Q water purification system (Millipore, Milford, MA, United States). Heparin sodium was purchased from Beijing Dingguo Changsheng Bio-Technology Co., Ltd. Other reagents used were of HPLC grade. Acetonitrile, methanol, and formic acid were purchased from Fisher Scientific (Fairlawn, NJ, United States).

### Animals

Male Sprague-Dawley rats, weighing 200 g ± 20 g (Certificate No. SCXK 2015-0004), were obtained from the Henan Experimental Animal Center (Zhengzhou, China). All animals were maintained on a 12/12 h light/dark cycle in an environmentally controlled breeding room (temperature 22°C ± 2°C; relative humidity 55% ± 5%). They were acclimated for one week before the initiation of dosing, with free access to food and water, and then fasted overnight (12 h) with water *ad libitum* prior to experimentation. The animal experiments were conducted in accordance with the National Institutes of Health Guide for the Care and Use of Laboratory Animals and approved by the Experimental Animal Ethics Committee of the Henan University of Chinese Medicine. A single 48.51 mg/kg dose of pinostrobin was separately administered orally to each group (pharmacokinetics, tissue distribution, and excretion groups). The samples from pharmacokinetics, tissue distribution, and excretion groups were used for metabolism studies.

### Pharmacokinetic Study

Pinostrobin solution was prepared in 0.9% sterile saline containing 2% polysorbate 80 (v/v). Pinostrobin was administered orally to five male rats. The volume of the dosing solution administered was adjusted according to the body weights recorded before dose administration. At 0 (prior to dosing), 0.133, 0.167, 0.33, 0.50, 1, 1.33, 1.67, 2, 4, 6, and 12 h after dosing, blood samples (∼400 µL) were collected from each animal via the fosse orbital vein using a heparinized 1.5 mL polythene tube. Plasma samples were obtained by immediate centrifugation (4,000 rpm for 10 min, 4°C) and stored at −80°C until analysis.

### Tissue Distribution Study

To investigate the tissue distribution of pinostrobin, rats were randomly divided into 11 groups of five rats each. Before and after oral administration of pinostrobin, the heart, liver, spleen, lung, kidney, stomach, small intestine, and large intestine samples were collected at 0 (prior to dosing), 0.083, 0.33, 0.75, 1, 2, 3, 6, 8, 12, and 24 h after dosing. Tissue samples were washed with normal saline and dried with filter paper. The chyme in the gastrointestinal tract (including the stomach, small intestine, and large intestine) was removed before washing. Subsequently, the samples were accurately weighed to obtain their wet weight and stored at −80°C until analysis.

### Excretion Study

Rats were randomly divided into two groups (*n* = 5). One group was used in the urinary and fecal excretion study. Five rats were orally administered pinostrobin and housed individually in stainless-steel metabolic cages, which allowed the separate collection of urine and feces. Urine and fecal samples were collected before administration and at time intervals of 0–2, 2–4, 4–6, 6–8, 8–12, 12–24, 24–36, and 36–54 h after dosing. The specimens were stored at −80°C after measuring the urine volume and the dry weight of feces for each time interval.

The other group was used in the biliary excretion study. Five rats were anesthetized and a cannula was implanted into the bile duct to collect bile. After the oral administration of pinostrobin, bile samples were collected at 0–3, 3–5, 5–7, 7–9, 9–12, 12–16, 16–20, and 20–24 h after dosing and stored at −80°C after recording the volume for each time interval.

### Instrument and Analytical Conditions

An ultra-high-performance liquid chromatography-linear trap quadrupole orbitrap mass spectrometry (UPLC-LTQ orbitrap-MS/MS) method was applied using a Dionex Ultimate 3000 UPLC tandem LTQ Orbitrap XL Hybrid mass spectrometer equipped with an electrospray ionization source (Thermo Fisher Scientific, Bremen, Germany). Chromatographic separation was performed on a Hypersil GOLD C18 column (2.1 mm × 50 mm, 1.9 μm) in tandem with a guard column and a UPLC filter cartridge (2.1 mm × 0.2 μm; Thermo Fisher Scientific) at a column temperature of 30°C. The mobile phase comprised 0.1% formic acid (v/v) aqueous solution (A) and acetonitrile (B). The elution gradient was set as follows: 0.0–15.0 min, 28.0–100.0% B; 15.0–15.1 min, 100.0–28.0% B; 15.1–18.0 min, 28% B. The flow rate was 0.3 mL/min, and the injection volume was 5 μL, with the autosampler conditioned at 7°C.

Mass spectrometry was performed in the positive ion mode. The ion source parameters were set as follows: capillary temperature, 350°C; ion spray voltage, 4.2 kV; capillary voltage, 49 V; tube lens voltage, 105 V; and the sheath (N_2_) and auxiliary gas (He) flow rates of 40 and 10 arbitrary units, respectively. For pharmacokinetics, tissue distribution, and excretion studies, selected ion monitoring (SIM) was used with the ion *m*/*z* 271.09607 indicating pinostrobin and *m*/*z* 257.08026 indicating isoliquiritigenin (Figure 1). For the metabolism study, full scanning was used with a scan range of *m*/*z* 80–1,500. The normalized collision energy for collision-induced dissociation (CID) was adjusted to 30% of the maximum. The isolation width of the precursor ions was *m*/*z* 2.0, and the default values were used for other CID parameters. Accurate masses were calibrated according to the manufacturer’s guidelines using a standard mixture of caffeine, MRFA, and Ultramark 1621. Data acquisition and processing were performed using Xcalibur 3.0 software (Thermo Fisher Scientific) and Mass Frontier 7.0 software (Thermo Fisher Scientific, Waltham, MA, United States) (Sun et al., 2016).

### Preparation of Standard and Quality Control Samples

The stock solutions of pinostrobin (1.0 mg/mL) and IS (0.4 mg/mL) were separately prepared in methanol and stored at 4°C. Standard solutions of pinostrobin at desired concentrations were prepared by serial dilution of the stock solution with methanol every 2 weeks. The IS solution was diluted with methanol to 400 ng/mL. All solutions were kept at 4°C before use. Calibration standards were prepared by spiking 20 µL of the appropriate standard solution, 50 µL IS solution, and 180 µL of blank rat plasma, urine, fecal, bile, or tissue homogenate samples. Calibration standards were prepared at concentrations of 4, 10, 50, 100, 500, 1,000, and 2,000 ng/mL for plasma, urine, fecal, and bile; 8, 20, 100, 200, 1,000, 2,000, and 4,000 ng/g for the heart, liver, spleen, lung, and kidney; 8, 20, 100, 200, 1,000, 2,000, 4,000, and 10,000 ng/g for the stomach, small intestine, and large intestine. The quality control (QC) samples included lower limit of quantification (LLOQ), low, middle, and high QCs. They were prepared in the same manner using blank biological samples of 4, 10, 400, and 2,000 ng/mL for plasma, urine, fecal, and bile samples; 8, 20, 800, and 4,000 ng/g for the heart, liver, spleen, lung, and kidney; and 8, 20, 1,000, and 10,000 ng/g for the stomach, small intestine, and large intestine, respectively. Calibration and QC samples were stored at 4°C until analysis.

### Sample Pretreatment

For the pharmacokinetic study, an aliquot of 200 µL of rat plasma samples (blank plasma, calibration standards, QC samples, and pharmacokinetic plasma samples) was vortexed with IS (50 µL, 400 ng/mL) and then with a solution of methanol–acetonitrile (1.0 mL, 5 : 95, v/v) for extraction. The mixture was vortexed for 5 min and then centrifuged at 10,000 rpm for 10 min at 4°C. The supernatant was transferred into a clean tube and evaporated to dryness under vacuum. The dried residue was reconstituted with 100 µL of methanol and vortexed at 2,000 rpm for 5 min. After centrifugation at 13,600 rpm for 10 min at 4°C, 5 µL of the supernatant was injected into the UPLC-LTQ orbitrap-MS/MS system for analysis. To study tissue distribution, each weighed tissue sample was homogenized in ice-cold physiological saline solution (1 : 2, w/v). The subsequent steps were identical to those described above for the treatment of plasma samples. For the excretion study, the fecal samples were pulverized with a mortar and pestle and homogenized in ice-cold physiological saline solution (1 : 2, w/v). Bile, urine, and homogenized fecal samples were processed using a similar method as the plasma samples.

### Method Validation

The method was validated for specificity, linearity, sensitivity, accuracy and precision, extraction recovery, and matrix effect according to the FDA guidance for industry on bioanalytical method validation. Specificity was assessed by comparing the chromatograms of the standard-spiked samples with the biosamples from six different sources. Calibration curves were plotted as the peak area ratio (drug/IS) versus the pinostrobin nominal concentration using weighted least-squares linear regression analysis. The lower limit of quantification was defined as the lowest concentration on the calibration curve and evaluated by analyzing the samples prepared in six replicates. Precision and accuracy were assessed with six replicates at four QC levels in three separate runs for three consecutive days using calibration curves that were established daily. Precision was expressed as the relative standard deviation (RSD, %), and accuracy was expressed as the relative error (RE %). Matrix effects are usually due to the influence of coeluting compounds on the actual analyte ionization process (Cappiello et al., 2008). Blank biosamples were used to evaluate the matrix effect at three QC levels (*n* = 6). The matrix effect was calculated by comparing the peak area ratio of the analyte relative to the IS in the analyte-spiked postextracted sample with that acquired using a neat solution (Zeng et al., 2019). Extraction recoveries of pinostrobin through the protein precipitation procedure were also evaluated at three QC concentrations (*n* = 6) and determined by comparing the peak areas obtained from blank biological matrices spiked with analyte before and after extraction.

### Metabolite Profiling

Metabolites were profiled using samples from the pharmacokinetics, tissue distribution, and excretion studies. Metabolite identification was performed using MetWorks software (Version 1.3 SP4, Thermo Fisher Scientific, San Jose, CA, United States) based on retention times, chemical composition, and fragmentation patterns on UPLC-LTQ orbitrap-MS/MS and compared with the available standards and literature to describe the metabolic profiles of pinostrobin in plasma, urine, feces, bile, and tissues.

### Data Analysis

The pharmacokinetics were calculated using Kinetica software (Version 5.1, Thermo Fisher Scientific, San Jose, CA, United States) with a noncompartmental statistical model. The peak concentration (C_max_) and time to reach C_max_ (T_max_) were obtained from actual data. The results are expressed as the mean ± standard deviation (SD).

## Results

### Method Validation

Owing to the high selectivity and specificity of the SIM mode, no significant endogenous interference was observed at the retention times of pinostrobin (7.17 min) and IS (3.50 min). The calibration curves for all analytes showed good linearity (*r* ^2^ > 0.9923) over the concentration ranges. Intra- and interday precisions for LLOQ were less than 20%, while those for low, middle, and high QC were within 15%. Intra- and interday accuracies for LLOQ were within −13.0% to 15.3%, while those for low, middle, and high QC were within −13.2% to 10.4%. Extraction recoveries ranged from 81.8% to 107.1%, with an RSD% less than 14.7%. Matrix effects ranged from 81.0% to 106.6%, with an RSD% less than 12.1%. These results (Supplementary Figures S2–S13 and Supplementary Tables S1–S3) indicated that the developed method was reproducible, accurate, and reliable for quantitative analysis of pinostrobin.

### Pharmacokinetics Study

**FIGURE 2:**
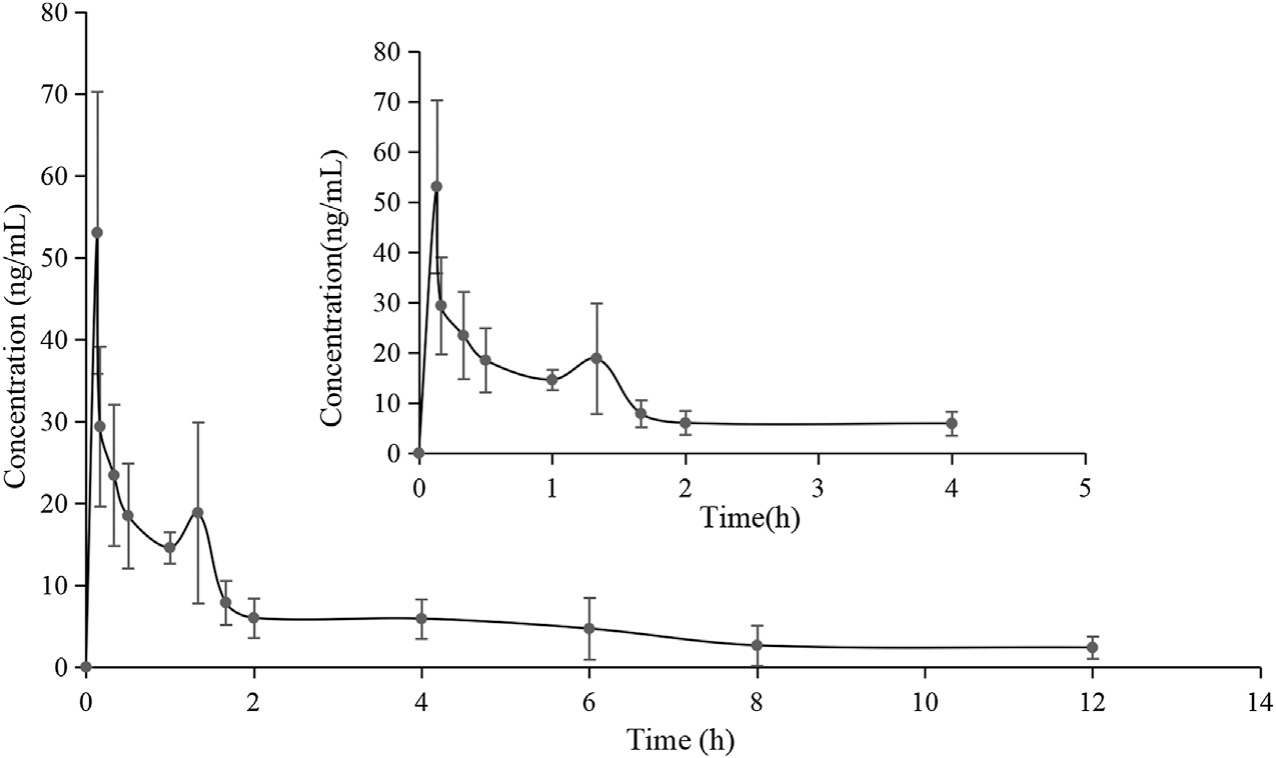
Mean plasma concentration-time curve of pinostrobin after oral administration of 48.51 mg/kg pinostrobin (*n* = 5, mean ± SD).

The validated method for the quantitation of pinostrobin in rat plasma was applied for the pharmacokinetic study in rats after oral administration of pinostrobin at a dose of 48.51 mg/kg. The mean plasma concentration-time curves are shown in Figure 2. The corresponding pharmacokinetic parameters calculated with noncompartmental analysis are listed as the mean ± SD and are shown in Table 1. The results showed that T_max_ was 0.133 h after oral administration in rats, and C_max_ of pinostrobin was 53.034 ng/mL ± 15.407 ng/mL. The apparent elimination half-life (t_1/2_) was 4.047 h ± 1.843 h, indicating that pinostrobin had a moderate t_1/2_. The mean residence time (MRT_0-∞_) was 5.906 h ± 2.056 h. The AUC_0-12h_ and AUC_0-∞_ were 721.659 ± 197.849 and 881.114 ± 289.587, respectively. The large apparent volume of distribution (V_z_) was 627.480 L/kg ± 111.057 L/kg.

**TABLE 1:** Noncompartmental pharmacokinetic parameters of pinostrobin in rats after oral administration of 48.51 mg/kg pinostrobin (*n* = 5, mean ± SD).

Pharmacokinetic parameters	Unit	Value
t_1/2_	h	4.047 ± 1.843
T_max_	h	0.133 ± 0
V_z_	L/kg	627.480 ± 111.057
CL	L/h/kg	126.278 ± 51.962
C_max_	ng/mL	53.034 ± 15.407
MRT_0-t_	h	3.437 ± 0.896
MRT_0-∞_	h	5.906 ± 2.056
AUC_0-24h_	ng/mL h	721.659 ± 197.849
AUC_0-∞_	ng/mL h	881.114 ± 289.587

### Tissue Distribution

**FIGURE 3:**
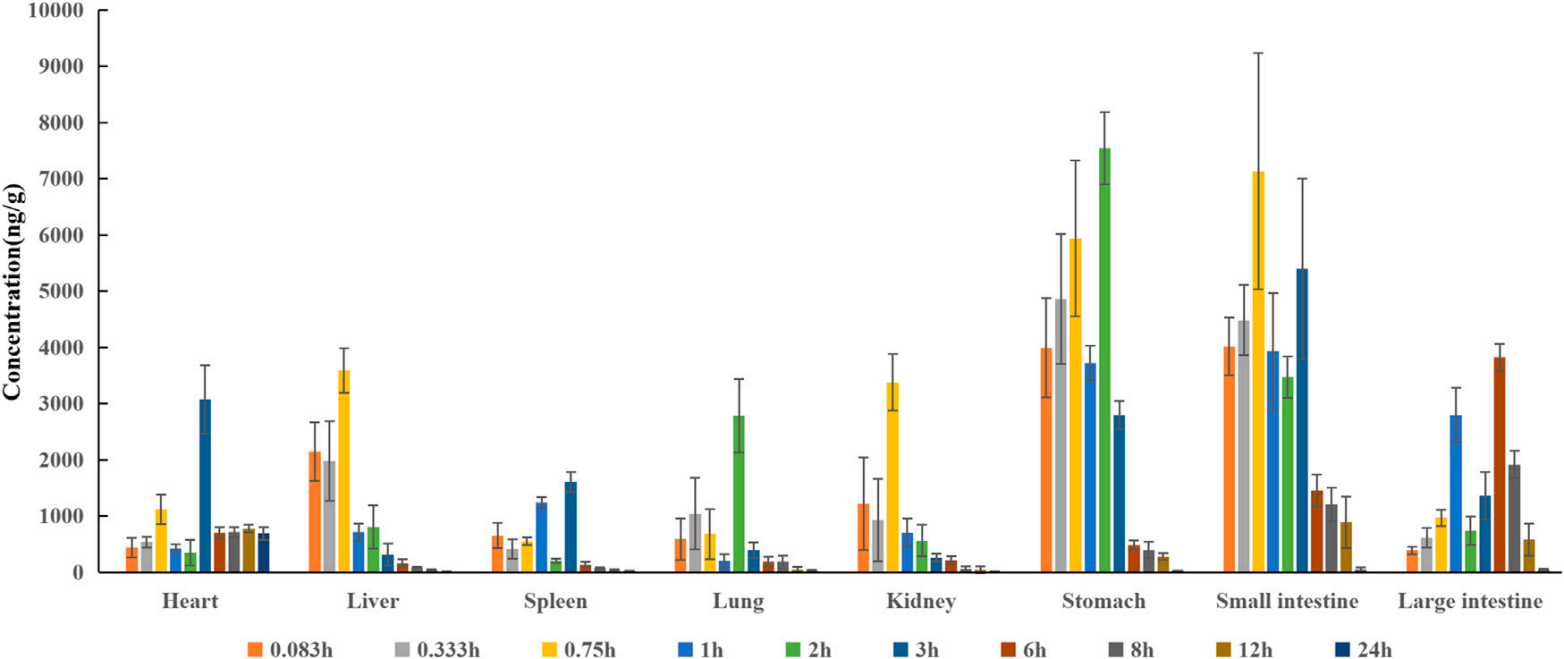
Tissue distribution profile of pinostrobin at different time points after a single oral administration of 48.51 mg/kg pinostrobin (*n* = 5, mean ± SD).

In the distribution study, the concentrations of pinostrobin were determined in multiple tissues within 24 h after a single oral administration. Tissue distribution profiles of pinostrobin in rats at different time points are shown in Figure 3. The C_max_, T_max_, and AUC for pinostrobin in tissues are shown in Supplementary Table S4. The results indicated that pinostrobin was widely distributed in the gastrointestinal tract and major organs. The highest concentration was observed in the stomach at 2 h, followed by the small intestine, large intestine, liver, kidney, heart, lung, and spleen. The accumulation of pinostrobin in tissues was in the order of small intestine > large intestine > stomach > heart > lung > liver > spleen > kidney, suggesting that the gastrointestinal tract may be the main target organ of pinostrobin. Combined with the previous pharmacological activities of antiulcer, anti-*HP*, anti-inflammatory, and antioxidation of pinostrobin, this suggests that it may be an effective component of traditional Chinese medicines for the treatment of peptic ulcer.

### Metabolite Profiling of Pinostrobin

**FIGURE 4:**
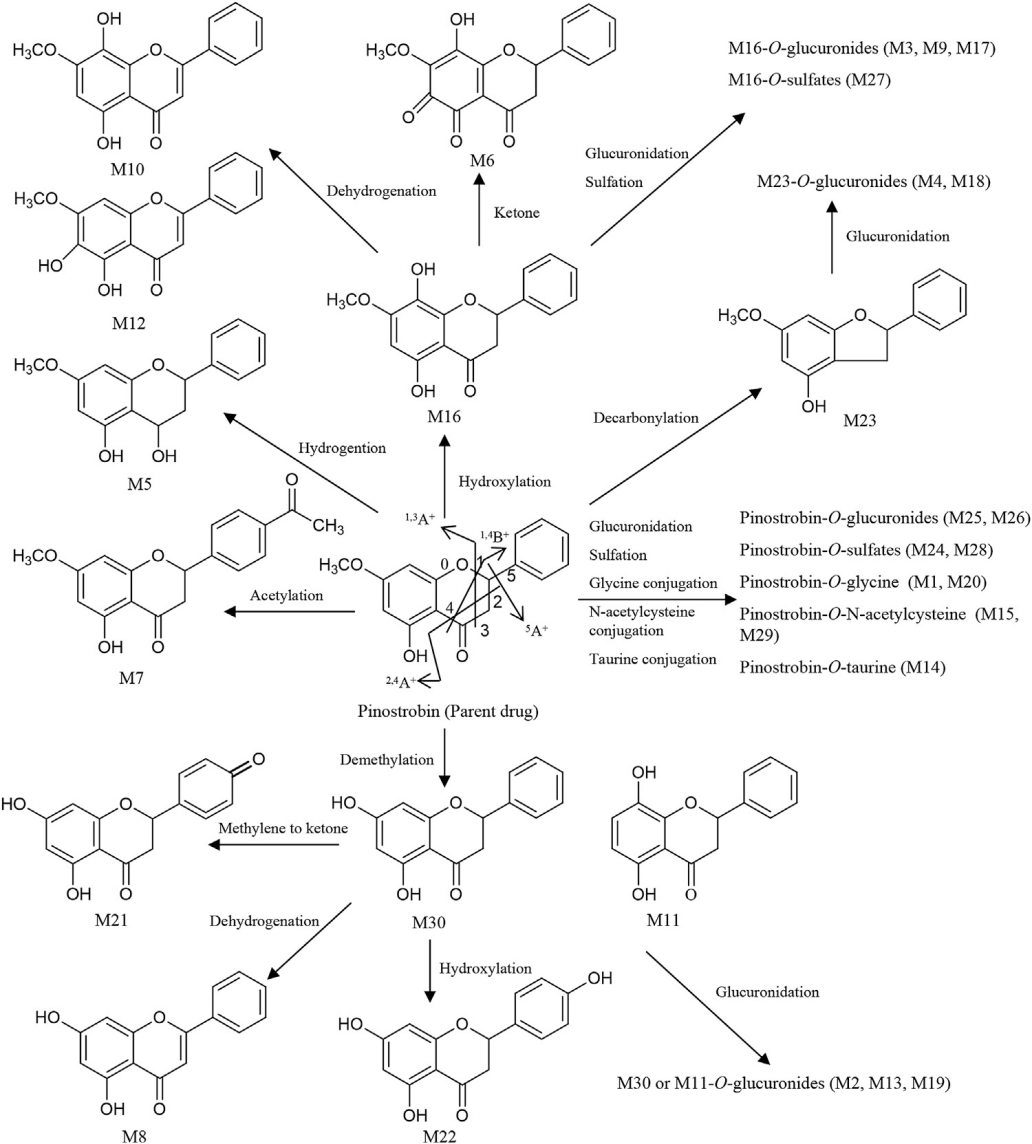
Proposed metabolic pathways of the metabolites in rats after oral administration of 48.51 mg/kg pinostrobin.

Thirty flavonoid metabolites were identified or partially identified in all biosamples. Their chromatograms are shown in Supplementary Figures S14–S17. The maximum peak area ratio of metabolites relative to the internal standard (R_max_) was used to estimate the content of these metabolites quantitatively. Their retention times, semiquantitative results, and fragment information are shown in Table 2. The proposed metabolic pathways of the above metabolites are illustrated in Figure 4. For these flavonoid metabolites, the subsequent loss of H_2_O, CO, and Retro Diels–Alder (RDA) reaction was observed in MS/MS fragmentation (Table 2), which is in line with reported results (Sun et al., 2016). A mass spectrum peak gave rise to [M + H]^+^ ions at *m*/*z* 271.09607 (C_16_H_15_O_4_) at a retention time of 7.14 min with the same fragmentation pathways as the pinostrobin standard, identified as the parent drug, pinostrobin. MS/MS fragmentation showed the ions at *m*/*z* 253.0854 (−18 Da, loss of H_2_O), ^2.4^A^**+**^ ions at *m*/*z* 229.0854, ^1.3^A^+^ ions at *m*/*z* 167.0333, ^1.4^B^+^ ions at *m*/*z* 131.0487, ^5^A^+^ ions at *m*/*z* 193.0488, and *m*/*z* 103.0539 (−28 Da, product of the fragment ions at *m*/*z* 131.0487 through the loss of CO) (Sun et al., 2016).

**TABLE 2:** The metabolites in rats after oral administration of 48.51 mg/kg pinostrobin.

Metabolites	RT (min)	[M + H]^+^	Chemical formula	ppm	Mass shift	Formula change	Metabolic pathway	MS^n^ m/z	Source (T_max_, R_max_)^a^
Parent drug	7.14	271.09607	C_16_H_15_O_4_	−1.532				253.08542[M + H-H_2_O]^+^, 229.08537[M + H-C_2_H_2_O]^+^, 193.04883[M + H-C_6_H_6_]^+^, 173.05914[M + H-C_2_H_2_O-C_3_H_4_O]^+^, 167.03336[M + H-C_8_H_8_]^+^, 131.04871[M + H-C_7_H_8_O_3_]^+^, 103.05387[M + H-C_7_H_8_O_3_-CO]^+^	Plasma, urine, feces, bile, heart, liver, spleen, lung, kidney, stomach, small intestine, and large intestine
M1	0.76	328.11810	C_18_H_18_O_5_N	0.460	57.0215	[M-OH + C_2_H_4_NO_2_]	Glycine conjugation	271.09689[M + H-C_2_H_3_NO]^+^, 167.03342[M + H-C_2_H_3_NO-C_8_H_8_]^+^, 131.04826[M + H-C_2_H_3_NO-C_7_H_8_O_3_]^+^, 103.05365[M + H-C_2_H_3_NO-C_7_H_8_O_3_-CO]^+^	Urine (8–12, 3.563), liver (0.083, 0.471), kidney (0.75, 0.075), and plasma (2, 0.011)
M2	0.84	433.11163	C_21_H_21_O_10_	−2.986	162.0164	[M-CH_2_ + C_6_H_8_O_6_]	Demethylation and glucuronidation	415.09778[M + H-H_2_O]^+^, 257.08044[M + H-C_6_H_8_O_6_]^+^, 131.04860[M + H-C_6_H_8_O_6_-C_6_H_6_O_3_]^+^	Urine (6–8, 2.671), bile (12–16, 0.539), small intestine (8, 0.090), lung (8, 0.062), and liver (0.33, 0.039)
M3	0.98	463.12338	C_22_H_23_O_11_	−0.233	192.0270	[M + C_6_H_8_O_7_]	Hydroxylation andglucuronidation	287.09042[M + H-C_6_H_8_O_6_]^+^, 269.07990[M + H-C_6_H_8_O_6_-H_2_O]^+^, 255.06409[M + H-C_6_H_8_O_6_-CH_4_O]^+^, 183.02962[M + H-C_6_H_8_O_6_-C_8_H_8_]^+^, 131.04849[M + H-C_6_H_8_O_6_-C_7_H_8_O_4_]^+^	Bile (16–20, 0.447); liver (0.33, 0.014)
M4	1.00	419.13272	C_21_H_23_O_9_	−2.240	148.0372	[M + C_5_H_8_O_5_]	Decarboxylation and glucuronidation	401.12317[M + H-H_2_O]^+^, 383.11270[M + H-_2_H_2_O]^+^, 243.10162[M + H-C_6_H_8_O_6_]^+^, 137.05962[M + H-C_6_H_8_O_6_-C_7_H_6_O]^+^, 133.06473[M + H-C_6_H_8_O_6_-C_6_H_6_O_2_]^+^, 123.04401[M + H-C_6_H_8_O_6_-C_8_H_8_O]^+^, 107.04910[M + H-C_6_H_8_O_6_-C_8_H_8_O_2_]^+^	Urine (12–24, 4.916), plasma (12, 0.040), kidney (12, 0.015), and lung (12, 0.011)
M5	1.04	273.11218	C_16_H_17_O_4_	0.163	2.0157	[M + H_2_]	Hydrogenation	243.10094[M + H-CH_2_O]^+^, 105.03316[M + H-C_11_H_12_O_3_]^+^, 91.05390[M + H-C_9_H_10_O_4_]^+^	Urine (8–12, 1.236), kidney (12, 0.077), and bile (0–3, 0.031)
M6	1.13	301.07001	C_16_H_13_O_6_	−2.174	29.9742	[M-H_2_ + O_2_]	Hydroxylation and ketone	271.05948[M + H-CH_2_O]^+^, 131.04871[M + H-CH_2_O-C_7_H_8_O_3_]^+^	Urine (24–36, 3.059), stomach (1, 0.016), heart (0.083, 0.014), plasma (1.67, 0.010), lung (24, 0.009), and spleen (24, 0.009)
M7	1.17	313.10632	C_18_H_17_O_5_	−2.332	42.0106	[M + COCH_2_]	Acetylation	295.09641[M + H-H_2_O]^+^, 277.08640[M + H-2H_2_O]^+^, 267.10165[M + H-H_2_O-CO]^+^, 253.08600[M + H-H_2_O-C_2_H_2_O]^+^, 249.09061[M + H-H_2_O-CO-H_2_O]^+^, 130.03491[M + H-C_9_H_11_O_4_]^+^	Feces (4–6, 12.513), large intestine (0.75, 0.423), urine (4–6, 0.352), stomach (0.75, 0.238), small intestine (0.083, 0.091), spleen (0.75, 0.031), and heart (12, 0.008)
M8	1.53	255.06461	C_15_H_11_O_4_	−2.256	−16.0308	[M-CH_2_-H_2_]	Demethylation and dehydrogenation	237.05391[M + H-H_2_O]^+^, 227.06953[M + H-CO]^+^, 209.05901[M + H-H2O-CO]^+^, 199.07465[M + H-2CO]^+^, 181.06406[M + H-2CO-H_2_O]^+^, 157.06416[M + H-2CO-C_2_H_2_O]^+^, 145.02780[M + H-C_6_H_6_O_2_]^+^, 137.02280[M + H-C_8_H_6_O]^+^	Urine (8–12, 108.404), small intestine (0.33, 8.065), stomach (0.33, 4.531), feces (2–4, 3.038), plasma (4, 0.504), kidney (12, 0.389), bile (0–3, 0.362), heart (0.33, 0.313), lung (0.33, 0.277), spleen (0.083, 0.257), liver (0.33, 0.115), and large intestine (0.33, 0.110)
M9	1.55	463.12256	C_22_H_23_O_11_	−2.004	192.0270	[M + C_6_H_8_O_7_]	Hydroxylation and glucuronidation	287.09058[M + H-C_6_H_8_O_6_]^+^, 269.08008[M + H-C_6_H_8_O_6_-H_2_O]^+^, 255.06412[M + H-C_6_H_8_O_6_-CH_4_O]^+^, 183.02753[M + H-C_6_H_8_O_6_-C_8_H_8_]^+^, 131.04784[M + H-C_6_H_8_O_6_-C_7_H_8_O_4_]^+^	Small intestine (8, 0.335), liver (0.33, 0.219), and kidney (0.75, 0.132)
M10	1.63	285.07507	C_16_H_13_O_5_	−2.385	13.9793	[M-H_2_ + O]	Hydroxylation and dehydrogenation	270.05151[M + H-CH_3_]^+^, 267.06436[M + H-H_2_O]^+^, 257.08008[M + H-CO]^+^, 229.08514[M + H-2CO]^+^, 225.05389[M + H-CO-CH_4_O]^+^, 197.05893[M + H-CO-CH_4_O-CO]^+^, 167.03322[M + H-C_8_H_6_O]^+^, 145.02779[M + H-C_7_H_8_O_3_]^+^, 137.02269[M + H-C_9_H_8_O_2_]^+^	Urine (24–36, 107.577), small intestine (0.33, 2.346), feces (8–12, 2.019), stomach (0.33, 1.195), plasma (4, 0.200), kidney (12, 0.115), lung (0.33, 0.090), heart (0.33, 0.077), bile (12–16, 0.067), spleen (0.33, 0.060), and large intestine (0.083, 0.030)
M11	1.93	257.08035	C_15_H_13_O_4_	−1.771	−14.0157	[M-CH_2_]	Demethylation	215.06979[M + H-C_2_H_2_O]^+^, 179.03320[M + H-C_6_H_6_]^+^, 173.05905[M + H-2C_2_H_2_O]^+^, 153.01764[M + H-C_8_H_8_]^+^, 131.04865[M + H-C_6_H_6_O_3_]^+^	Urine (0–2, 6.840), feces (36–54, 1.937), plasma (6, 0.674), bile (12–16, 0.221), stomach (12, 0.186), kidney (12, 0.153), liver (6, 0.121), heart (2, 0.121), spleen (24, 0.112), large intestine (0.75, 0.109), lung (1, 0.108), and small intestine (24, 0.103)
M12	1.95	285.07562	C_16_H_13_O_5_	−0.456	13.9793	[M-H_2_ + O]	Hydroxylation anddehydrogenation	257.07993[M + H-CO]^+^, 167.03354[M + H-C_8_H_6_O]^+^	Urine (8–12, 30.236); bile (0–3, 0.305)
M13	1.99	433.11176	C_21_H_21_O_10_	−2.686	162.0164	[M-CH_2_ + C_6_H_8_O_6_]	Demethylation and glucuronidation	257.07996[M + H-C_6_H_8_O_6_]^+^, 215.06911[M + H-C_6_H_8_O_6_-C_2_H_2_O]^+^, 153.01761[M + H-C_6_H_8_O_6_-C_8_H_8_]^+^, 131.04823[M + H-C_6_H_8_O_6_-C_6_H_6_O_3_]^+^, 103.05369[M + H-C_6_H_8_O_6_-C_6_H_6_O_3_-CO]^+^	Bile (16–20, 0.647), small intestine (8, 0.204), and urine (12–24, 0.114)
M14	2.07	378.10001	C_18_H_20_O_6_NS	−1.519	107.0041	[M + C_2_H_5_NO_2_S]	Taurine conjugation	271.09558[M + H-C_2_H_5_NO_2_S]^+^, 167.03336[M + H-C_2_H_5_NO_2_S-C_8_H_8_]^+^, 131.04861[M + H-C_2_H_5_NO_2_S-C_7_H_8_O_3_]^+^	Urine (0–2, 0.502), heart (3–6, 0.146), spleen (0.75, 0.071), plasma (4, 0.053), small intestine (3, 0.027), and stomach (0.33, 0.008)
M15	2.18	432.11047	C_21_H_22_O_7_NS	−1.572	161.0147	[M + C_5_H_7_NO_3_S]	*N*-Acetylcysteine conjugation	399.13025[M + H-HS]^+^, 381.11737[M + H-HS-H_2_O]^+^, 271.09607[M + H-C_5_H_7_NO_3_S]^+^, 173.05907[M + H-C_5_H_7_NO_3_S-C_2_H_2_O-C_3_H_4_O]^+^, 167.03336[M + H-C_5_H_7_NO_3_S-C_8_H_8_]^+^, 131.04878[M + H-C_5_H_7_NO_3_S-C_7_H_8_O_3_]^+^, 103.05384[M + H-C_5_H_7_NO_3_S-C_7_H_8_O_3_-CO]^+^	Urine (0–2, 0.792)
M16	2.24	287.09088	C_16_H_15_O_5_	−1.811	15.9949	[M + O]	Hydroxylation	269.08017[M + H-H_2_O]^+^, 255.06470[M + H-CH_4_O]^+^, 245.08011[M + H-C_2_H_2_O]^+^, 183.02817[M + H-C_8_H_8_]^+^, 173.05904[M + H-C_2_H_2_-C_3_H_4_O_2_]^+^, 131.04868[M + H-C_7_H_8_O_4_]^+^, 103.05385[M + H-C_7_H_8_O_4_-CO]^+^	Urine (0–2, 59.881), bile (0–3, 21.765), small intestine (3, 3.497), plasma (6, 3.053), kidney (0.75, 1.180), liver (3, 0.321), lung (0.083, 0.082), heart (0.33, 0.072), and stomach (0.33, 0.038)
M17	2.35	463.12259	C_22_H_23_O_11_	−1.939	192.0270	[M + C_6_H_8_O_7_]	Hydroxylation and glucuronidation	445.11160[M + H-H_2_O]^+^, 427.10352[M + H-2H_2_O]^+^, 287.09064[M + H-C_6_H_8_O_6_]^+^, 269.08014[M + H-C_6_H_8_O_6_-H_2_O]^+^, 255.06468[M + H-C_6_H_8_O_6_-CH_4_O]^+^, 245.08002[M + H-C_6_H_8_O_6_-C_2_H_2_O]^+^, 183.02814[M + H-C_6_H_8_O_6_-C_8_H_8_]^+^, 173.05910[M + H-C_6_H_8_O_6_-C_2_H_2_-C_3_H_4_O_2_]^+^, 131.04861[M + H-C_6_H_8_O_6_-C_7_H_8_O_4_]^+^, 103.05376[M + H-C_6_H_8_O_6_-C_7_H_8_O_4_-CO]^+^	Urine (0–2, 109.471), bile (0–3, 31.084), plasma (6, 6.648), small intestine (3, 5.256), kidney (0.75, 1.794), liver (3, 0.457), heart (0.33, 0.135), stomach (8, 0.116), lung (0.083, 0.112), and spleen (6, 0.016)
M18	2.36	419.13333	C_21_H_23_O_9_	−0.784	148.0372	[M + C_5_H_8_O_5_]	Decarboxylation and glucuronidation	243.10045[M + H-C_6_H_8_O_6_]^+^, 137.05922[M + H-C_6_H_8_O_6_-C_7_H_6_O]^+^, 123.04356[M + H-C_6_H_8_O_6_-C_8_H_8_O]^+^, 107.04873[M + H-C_6_H_8_O_6_-C_8_H_8_O_2_]^+^	Urine (2–4, 0.546)
M19	2.45	433.11200	C_21_H_21_O_10_	−2.132	162.0164	[M-CH_2_ + C_6_H_8_O_6_]	Demethylation and glucuronidation	415.10141[M + H-H_2_O]^+^, 397.09091[M + H-2H_2_O]^+^, 257.08026[M + H-C_6_H_8_O_6_]^+^, 239.06920[M + H-C_6_H_8_O_6_-H_2_O]^+^, 215.06953[M + H-C_6_H_8_O_6_-C_2_H_2_O]^+^, 179.03311[M + H-C_6_H_8_O_6_-C_6_H_6_]^+^, 173.05902[M + H-C_6_H_8_O_6_-2C_2_H_2_O]^+^, 153.01761[M + H-C_6_H_8_O_6_-C_8_H_8_]^+^, 145.06438[M + H-C_6_H_8_O_6_-2C_2_H_2_O-CO]^+^, 131.04861[M + H-C_6_H_8_O_6_-C_6_H_6_O_3_]^+^, 103.05379[M + H-C_6_H_8_O_6_-C_6_H_6_O_3_-CO]^+^	Urine (0–2, 203.526), plasma (4, 25.663), bile (0–3, 18.171), kidney (0.75, 4.648), small intestine (3, 3.140), heart (0.75, 0.420), liver (3, 0.241), lung (0.083, 0.186), stomach (8, 0.158), and spleen (2, 0.009)
M20	2.57	328.11697	C_18_H_18_O_5_N	−2.984	57.0215	[M-OH + C_2_H_4_NO_2_]	Glycine conjugation	271.09583[M + H-C_2_H_3_NO]^+^, 167.03299[M + H-C_2_H_3_NO-C_8_H_8_]^+^, 131.04858[M + H-C_2_H_3_NO-C_7_H_8_O_3_]^+^	Bile (3–5, 0.509), urine (8–12, 0.461), small intestine (0.75, 0.298), plasma (4, 0.165), spleen (0.75, 0.103), liver (3, 0.102), stomach (24, 0.069), kidney (0.75, 0.041), large intestine (8, 0.033), heart (3, 0.027), and lung (8, 0.020)
M21	2.64	271.05951	C_15_H_11_O_5_	−2.176	−0.0364	[M-CH_4_ + O]	Demethylation and methylene to ketone	253.04883[M + H-H_2_O]+, 243.06450[M + H-CO]+, 229.04872[M + H-C_2_H_2_O]+, 225.05394[M + H-CO-H_2_O]^+^, 215.06960[M + H-2CO]^+^, 197.05894[M + H-2CO-H_2_O]^+^, 187.03836[M + H-2C_2_H_2_O]^+^, 169.06418[M + H-3CO-H_2_O]^+^, 159.04343[M + H-2C_2_H_2_O-CO]^+^, 153.01765[M + H-C_8_H_6_O]+, 149.02278[M + H-C_7_H_6_O_2_]+, 145.02782[M + H-C_8_H_6_O_3_]^+^, 119.04878[M + H-C_7_H_4_O_4_]^+^	Urine (24–36, 200.177), small intestine (0.33, 5.512), stomach (0.33, 4.903), feces (2–4, 2.119), liver (0.33, 0.425), heart (0.33, 0.365), lung (0.33, 0.271), spleen (0.75, 0.221), plasma (4, 0.179), large intestine (8, 0.108), and spleen (6, 0.064)
M22	2.72	273.07455	C_15_H_13_O_5_	−4.394	1.9793	[M-CH_2_ + O]	Demethylation and hydroxylation	153.01706[M + H-C_8_H_8_O]^+^, 121.06379[M + H-C_7_H_4_O_4_]^+^	Feces (8–12, 1.467), liver (0.33, 1.427), small intestine (6, 1.101), large intestine (8, 0.483), urine (4–6, 0.457), stomach (0.75, 0.249), kidney (0.75, 0.243), spleen (0.75, 0.220), lung (0.75, 0.134), and heart (2, 0.021)
M23	2.77	243.10080	C_15_H_15_O_3_	−3.171	−27.9949	[M-CO]	Decarboxylation	137.05891[M + H-C_7_H_6_O]^+^, 133.06447[M + H-C_6_H_6_O_2_]^+^, 123.04386[M + H-C_8_H_8_O]^+^, 107.04888[M + H-C_8_H_8_O_2_]^+^	Urine (36–54, 136.900), feces (0–2, 0.270), large intestine (2, 0.222), stomach (6, 0.179), small intestine (12, 0.100), liver (2, 0.059), kidney (12, 0.052), spleen (6, 0.038), and lung (12, 0.020)
M24	2.82	351.05319	C_16_H_15_O_7_S	−0.313	79.9568	[M + SO_3_]	Sulfation	271.09619[M + H-SO_3_]^+^, 253.08521[M + H-SO_3_-H_2_O]^+^, 229.08615[M + H-SO_3_-C_2_H_2_O]^+^, 193.04921[M + H-SO_3_-C_6_H_6_]^+^, 173.05945[M + H-SO_3_-C_2_H_2_O-C_3_H_4_O]^+^, 167.03351[M + H-SO_3_-C_8_H_8_]^+^, 131.04875[M + H-SO_3_-C_7_H_8_O_3_]^+^, 103.05399[M + H-SO_3_-C_7_H_8_O_3_-CO]^+^	Plasma (2, 0.119); urine (0–2, 0.064)
M25	2.94	447.12799	C_22_H_23_O_10_	−1.305	176.0321	[M + C_6_H_8_O_6_]	Glucuronidation	429.11697[M + H-H_2_O]^+^, 411.10663[M + H-2H_2_O]^+^, 313.10654[M + H-C_4_H_6_O_5_]^+^, 271.09592[M + H-C_6_H_8_O_6_]^+^, 253.08542[M + H-C_6_H_8_O_6_-H_2_O]^+^, 229.08516[M + H-C_6_H_8_O_6_-C_2_H_2_O]^+^, 193.04890[M + H-C_6_H_8_O_6_-C_6_H_6_]^+^, 173.05908[M + H-C_6_H_8_O_6_-C_2_H_2_O-C_3_H_4_O]^+^, 167.03326[M + H-C_6_H_8_O_6_-C_8_H_8_]^+^, 131.04866[M + H-C_6_H_8_O_6_-C_7_H_8_O_3_]^+^, 103.05376[M + H-C_6_H_8_O_6_-C_7_H_8_O_3_-CO]^+^	Urine (0–2, 406.932), bile (0–3, 174.894), plasma (0.133, 59.330), small intestine (3, 25.059), liver (0.083, 7.034), kidney (0.75, 7.225), stomach (0.33, 1.090), heart (0.33, 0.967), lung (0.083, 0.538), and spleen (0.33, 0.039)
M26	3.09	447.12772	C_22_H_23_O_10_	−1.908	176.0321	[M + C_6_H_8_O_6_]	Glucuronidation	429.11682[M + H-H_2_O]^+^, 411.10645[M + H-2H_2_O]^+^, 313.10657[M + H-C_4_H_6_O_5_]^+^, 271.09586[M + H-C_6_H_8_O_6_]^+^, 253.08565[M + H-C_6_H_8_O_6_-H_2_O]^+^, 229.08559[M + H-C_6_H_8_O_6_-C_2_H_2_O]^+^, 193.04910[M + H-C_6_H_8_O_6_-C_6_H_6_]^+^, 173.05930[M + H-C_6_H_8_O_6_-C_2_H_2_O-C_3_H_4_O]^+^, 167.03351[M + H-C_6_H_8_O_6_-C_8_H_8_]^+^, 131.04883[M + H-C_6_H_8_O_6_-C_7_H_8_O_3_]^+^, 103.05399[M + H-C_6_H_8_O_6_-C_7_H_8_O_3_-CO]^+^	Urine (0–2, 385.560), bile (0–3, 221.411), plasma (0.133, 92.104), small intestine (3, 35.601), liver (0.083, 14.678), kidney (0.75, 10.093), stomach (0.33, 1.536), heart (0.33, 1.508), lung (0.083, 0.767), and spleen (0.33, 0.086)
M27	3.14	367.04578	C_16_H_15_O_8_S	−6.633	95.9517	[M + O_4_S]	Hydroxylation and sulfation	287.09122[M + H-SO_3_]^+^, 269.08093[M + H-SO_3_-H_2_O]^+^, 255.06427[M + H-SO_3_-CH_4_O]^+^, 131.04861[M + H-SO_3_-C_7_H_8_O_4_]^+^	Plasma (0.167, 0.157), urine (0–2, 0.134), liver (0.33, 0.023), kidney (0.33, 0.022), and lung (0.083, 0.006)
M28	4.08	351.05292	C_16_H_15_O_7_S	−1.082	79.9568	[M + SO_3_]	Sulfation	271.09613[M + H-SO_3_]+, 253.08557[M + H-SO_3_-H_2_O]^+^, 229.08524[M + H-SO_3_-C_2_H_2_O]^+^, 193.04897[M + H-SO_3_-C_6_H_6_]^+^, 173.05910[M + H-SO_3_-C_2_H_2_O-C_3_H_4_O]^+^, 167.03342[M + H-SO_3_-C_8_H_8_]^+^, 131.04875[M + H-SO_3_-C_7_H_8_O_3_]^+^, 103.05390[M + H-SO_3_-C_7_H_8_O_3_-CO]^+^	Plasma (0.167, 1.786); urine (0–2, 0.554)
M29	4.55	432.11029	C_21_H_22_O_7_NS	1.988	161.0147	[M + C_5_H_7_NO_3_S]	*N*-Acetylcysteine conjugation	271.09592[M + H-C_5_H_7_NO_3_S]^+^, 173.05887[M + H-C_5_H_7_NO_3_S-C_2_H_2_O-C_3_H_4_O]^+^, 167.03326[M + H-C_5_H_7_NO_3_S-C_8_H_8_]^+^, 131.04874[M + H-C_5_H_7_NO_3_S-C_7_H_8_O_3_]^+^	Urine (0–2, 0.289)
M30	5.01	257.07980	C_15_H_13_O_4_	−4.028	−14.0157	[M-CH_2_]	Demethylation	239.07014[M + H-H_2_O]+, 215.06995[M + H-C_2_H_2_O]^+^, 179.03355[M + H-C_6_H_6_]^+^, 173.05936[M + H-2C_2_H_2_O]^+^, 153.01790[M + H-C_8_H_8_]^+^, 145.06441[M + H-2C_2_H_2_O-CO]^+^, 131.04884[M + H-C_6_H_6_O_3_]+,103.05395[M + H-C_6_H_6_O_3_-CO]^+^	Urine (4–6, 17.160), small intestine (0.75, 15.426), liver (0.33, 11.587), large intestine (8, 9.070), kidney (0.75, 6.286), feces (12, 4.799), stomach (0.75, 2.849), spleen (0.75, 1.897), lung (0.75, 1.772), and heart (0.33, 0.858)

T_max_ is the time or time interval to reach the maximum peak area ratio of metabolites relative to the internal standard in biological samples. R_max_ is the maximum peak area ratio of metabolites relative to the internal standard.

a Sorted by R_max_ from large to small.

Metabolites M25 and M26 eluted at 2.94 min and 3.09 min, respectively, with the same [M + H]^+^ ions at *m*/*z* 447.12780 (C_22_H_23_O_10_), 176 Da higher than pinostrobin. The prominent fragment ion at *m*/*z* 271.0961 (C_16_H_15_O_4_) was obtained by losing a terminal glucose group (C_6_H_8_O_6_). The other product ions were similar to those of pinostrobin, indicating glucuronidation of pinostrobin. Metabolites M24 and M28 eluted at 2.82 min and 4.08 min, respectively, with the same [M + H]^+^ ions at *m*/*z* 351.0532 (C_16_H_15_O_7_S), 80 Da higher than pinostrobin. The neutral loss of a sulfate group (SO_3_) yielded the major fragment ion at *m*/*z* 271.0962 (C_16_H_15_O_4_), and the other ions at *m*/*z* 253.0852 and ^2.4^A^**+**^ ions at *m*/*z* 229.0861, ^1.3^A^+^ ions at *m*/*z* 167.0335, ^1.4^B^+^ ions at *m*/*z* 131.0487, and ^5^A^+^ ions at *m*/*z* 193.0492 and *m*/*z* 103.0540 were also observed, indicating sulfation of pinostrobin. Metabolites M1 and M20 had a retention time of 0.76 min and 2.57 min, respectively, with the same [M + H]^+^ ions at *m*/*z* 328.1181 (C_18_H_18_O_5_N), 57 Da higher than pinostrobin. The major fragment ion at *m*/*z* 271.0969 (C_16_H_15_O_4_) was produced by losing a glycine group (C_2_H_3_NO), and the other fragment ions at 167.0334 and 131.0483 were similar to those of pinostrobin, indicating glycine conjugation of pinostrobin. Metabolites M15 and M29 eluted at 2.18 and 4.55 min, respectively, with the same [M + H]^+^ ions at *m*/*z* 432.1105 (C_21_H_22_O_7_NS), 161 Da higher than pinostrobin. The fragment ion at *m*/*z* 271.0969 (C_16_H_15_O_4_) was due to the loss of a glycine group (C_5_H_7_NO_3_S). The other fragment ions at 167.0333 and 131.0486 were similar to those of pinostrobin, suggesting glycine conjugation of pinostrobin. Metabolite M14 showed [M + H]^+^ ions at *m*/*z* 378.1000 (C_18_H_20_O_6_NS), with a retention time of 2.07 min. The fragment ion at *m*/*z* 271.0956 (C_16_H_15_O_4_) was produced through the loss of a taurine group (C_2_H_5_NO_2_S, −107 Da), and mass signals at 167.0334 and 131.0486 were produced by ^1.3^A^+^ and ^1.4^B^+^ ions, which were from the ions at *m*/*z* 271.0956, indicating taurine conjugation of pinostrobin.

Metabolite M23 eluted at 2.77 min, with the [M + H]^+^ ions at *m*/*z* 243.1008 (C_15_H_15_O_3_), 28 Da (CO) lower than pinostrobin. The signals at *m*/*z* 123.0439, 107.0489, and 137.0589 demonstrated the existence of ^0.3^A^+^, ^0.2^B^+^, and ^0.2^A^+^ fragment ions, suggesting decarbonylation on the C ring. Metabolites M4 and M18 showed the same [M + H]^+^ ions at *m*/*z* 419.1327 (C_21_H_23_O_9_), with a retention time of 1.00 and 2.36 min, respectively, which was 176 Da higher than M23. The prominent fragment ion at *m*/*z* 243.1016 (C_15_H_15_O_3_) was obtained through the loss of a glucose group (C_6_H_8_O_6_). The other product ions were similar to that of M23, indicating decarbonylation and glucuronidation of pinostrobin.

Metabolite M16, with a retention time of 2.24 min, generated [M + H]^+^ ions at *m*/*z* 287.0908 (C_16_H_15_O_5_), which was 16 Da higher than that of pinostrobin. The product ion at *m*/*z* 269.0802 was obtained through the loss of H_2_O. The RDA reaction of M16 generated ^2.4^A^+^ ions at *m*/*z* 245.0801, ^1.3^A^+^ ions at *m*/*z* 183.0282, and ^1.4^B^+^ ions at *m*/*z* 131.0489. The fragment ions at *m*/*z* 131.0489 could be fragmented to produce the ion at *m*/*z* 103.0539 through the loss of CO. The fragment ion at *m*/*z* 183.0282 and 131.0488 showed hydroxylation on the A ring. Metabolites M10 and M12 eluted at 1.63 min and 1.95 min, respectively, with the same [M + H]^+^ ions at *m*/*z* 285.0756 (C_16_H_13_O_5_), 2 Da lower than M16. The fragment ions at *m*/*z* 167.0333 and 145.0277 demonstrated the existence of ^1.3^A^+^ and ^0.4^B^+^ fragment ions, suggesting hydroxylation of M16 on the C ring, which indicates the hydroxylation and dehydrogenation of pinostrobin. Metabolite M6 eluted at 1.13 min, with the [M + H]^+^ ions at *m*/*z* 301.0700 (C_16_H_13_O_6_), 14 Da (−2H + O) lower than that of pinostrobin. The signals at *m*/*z* 271.05948 and 131.04871 suggested hydroxylation and a ketone on the A ring of pinostrobin. Metabolites M3, M9, and M17 showed the same [M + H]^+^ ions at *m*/*z* 463.1225 (C_22_H_23_O_11_), with retention times of 0.98, 1.55, and 2.35 min, respectively, which was 176 Da higher than M16. The prominent fragment ion at *m*/*z* 287.0906 (C_16_H_15_O_5_) was obtained through the loss of a glucose group (C_6_H_8_O_6_). The other product ions were similar to that of M16, indicating hydroxylation and glucuronidation of pinostrobin. Metabolite M27 had a retention time of 3.14 min, with the [M + H]^+^ ions at *m*/*z* 367.0458 (C_16_H_15_O_8_S), 80 Da higher than M16. The neutral loss of a sulfate group (SO_3_) yielded the major fragment ion at *m*/*z* 287.0912 (C_16_H_15_O_5_). The other ions at *m*/*z* 269.0809 and 131.0486 were also observed, indicating hydroxylation and sulfation of pinostrobin.

The metabolite M30 (pinocembrin), with a retention time of 5.01 min, generated [M + H]^+^ ions at *m*/*z* 257.0798 (C_15_H_13_O_4_), indicating demethylation of pinostrobin. The fragment ion at *m*/*z* 239.0701 (C_7_H_5_O_4_) was obtained through the loss of H_2_O. The RDA reaction of M30 generated ^2.4^A^+^ ions at *m*/*z* 215.0699 (C_13_H_11_O_3_), ^1.3^A^+^ ions at *m*/*z* 153.0179 (C_7_H_5_O_4_), and ^1.4^B^+^ ions at *m*/*z* 131.0488 (C_9_H_7_O). The fragment ions at *m*/*z* 131.0488 could be fragmented to produce the ion at *m*/*z* 103.0540 (C_8_H_7_) through the loss of CO. The fragment ion at *m*/*z* 153.0179 and 131.0488 indicated demethylation on the A ring. The metabolite M11 generated the same [M + H]^+^ ions at 257.0804 (C_15_H_13_O_4_) and fragment ions at *m*/*z* 215.0698, 153.0176, and 131.0487 following an RDA reaction, which was identified as the isomer of M30. Metabolites M2, M13, and M19 showed the same [M + H]^+^ ions at *m*/*z* 463.1225 (C_22_H_23_O_11_), with a retention time of 0.84 min, 1.99 min, and 2.45 min, respectively, which was 176 Da higher than M30 or M11. The prominent fragment ion at *m*/*z* 257.0803 (C_15_H_13_O_4_) was obtained through the loss of a glucose group (C_6_H_8_O_6_). The other product ions were similar to those of M30 and M11, indicating demethylation and glucuronidation of pinostrobin. Metabolite M21 eluted at 2.64 min, with the [M + H]^+^ ions at *m*/*z* 271.0595 (C_15_H_11_O_5_), 14 Da (−2H + O) lower than M30. The fragment ions at *m*/*z* 153.0177, 119.0487, and 145.0278 demonstrated the existence of ^1.3^A^+^, ^1.3^B^+^, and ^1.4^B^+^ fragment ions, suggesting conversion of the methylene to a ketone of M30 on the B ring, indicating the demethylation and methylene conversion to the ketone of pinostrobin. Metabolite M8 eluted at 1.53 min, with the [M + H]^+^ ions at *m*/*z* 255.0646 (C_15_H_11_O_4_), 2 Da lower than M30. The mass signals at *m*/*z* 145.0277 and 137.0228 demonstrated the existence of ^0.4^B^+^ and ^0.3^B^+^ fragment ions, suggesting dehydrogenation of M30 on the C ring, indicating the demethylation and dehydrogenation of pinostrobin. Metabolite M22 with a retention time of 2.72 min generated [M + H]^+^ ions at *m*/*z* 273.0746 (C_15_H_13_O_5_), 16 Da higher than M30. The characteristic fragment ions ^1.3^A^+^ ions at *m*/*z* 153.0171 and ^1.3^B^+^ ions at *m*/*z* 121.0638 showed hydroxylation of M30 on the B ring, suggesting the demethylation and hydroxylation of pinostrobin.

Metabolite M7 had a retention time of 1.17 min and showed [M + H]^+^ ions at *m*/*z* 313.1063 (C_18_H_17_O_5_), 42 Da (COCH_2_) higher than pinostrobin. The fragment ion at *m*/*z* 130.03491 showed acetylation on the B ring. Metabolite M5 eluted at 1.04 min, with the [M + H]^+^ ions at *m*/*z* 273.1121 (C_16_H_17_O_4_), 2 Da higher than that of pinostrobin. The signals at *m*/*z* 91.0539 demonstrated the ^1.2^B^+^ fragment ions, suggesting hydrogenation on the C ring.

**FIGURE 5:**
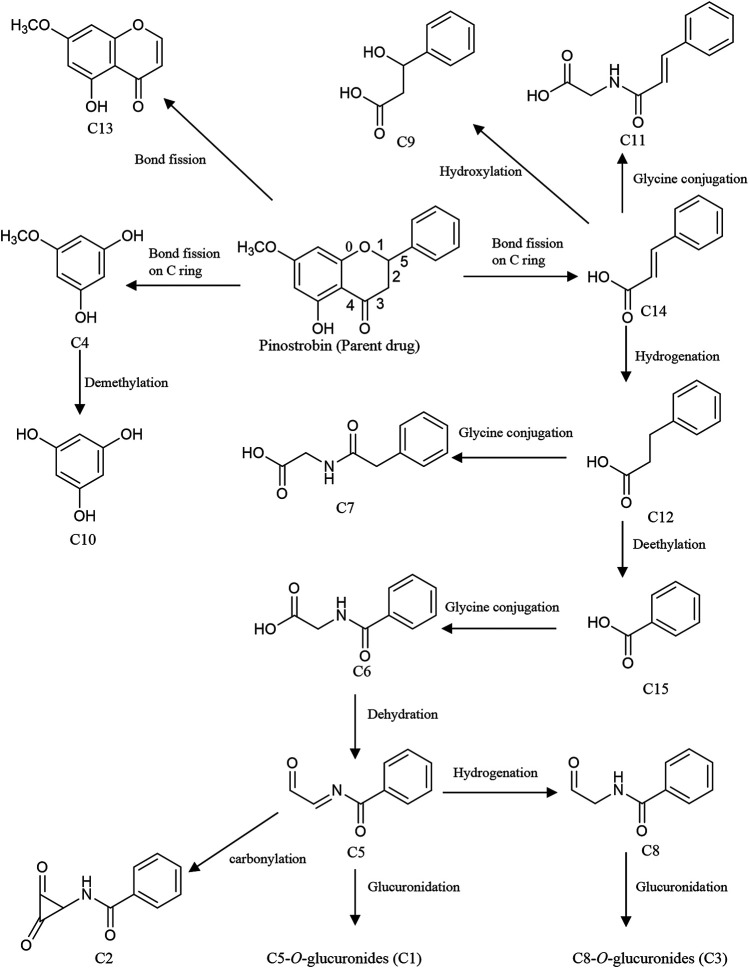
Proposed pathways of the catabolic metabolites by intestinal bacteria in rats after oral administration of 48.51 mg/kg pinostrobin.

Meanwhile, intestinal bacteria play an essential role in the metabolism of flavonoids, generating ring fission products (Liu et al., 2012; Zeng et al., 2018). In this study, 15 catabolites were detected (Table 3). Their chromatograms are shown in Supplementary Figures S18, S19. Pinostrobin was catabolized into C13 (C_10_H_9_O_4_) by fission of the fifth bond as well as C4 (C_6_H_7_O_3_) and C14 (C_9_H_9_O_2_) by the fission of the first and fourth bonds. C4 was converted to C10 by demethylation. Meanwhile, C14 was hydroxylated, hydrogenated, and glycine-conjugated, yielding C9 (C_9_H_11_O_3_), C12 (C_9_H_11_O_2_), and C11 (C_11_H_12_O_3_N). Then, C12 was glycine-conjugated and deethylated, generating C7 (C_10_H_12_O_3_N) and C15 (C_7_H_7_O_2_). C15 was glycine-conjugated to yield C6 (C_9_H_10_O_3_N). The dehydration of C6 produced C5. In addition, C5 could be hydrogenated, carbonylated, and glucuronidated, yielding C8 (C_9_H_10_O_2_N), C2 (C_10_H_8_O_3_N), and C1 (C_15_H_16_O_8_N). C6 was further converted to C3 by glucuronidation. Proposed pathways of the catabolic metabolites of pinostrobin are illustrated in Figure 5.

**TABLE 3:** The catabolic metabolites in rats after oral administration of 48.51 mg/kg pinostrobin.

Metabolites	RT (min)	[M + H]^+^	Chemical formula	ppm	MS^n^ m/z	Source (T_max_, R_max_)^a^
C1	0.70	338.08725	C_15_H_16_O_8_N	−1.902	320.07602[M + H-H_2_O]^+^, 162.05435[M + H-C_6_H8O_6_]^+^, 144.04382[M + H-C_6_H_8_O_6_-H_2_O]^+^, 116.04903[M + H-C_6_H8O_6_-H_2_O-CO]^+^	Urine (36–54, 23.329); feces (4–6, 0.076)
C2	0.69	190.04930	C_10_H_8_O_3_N	−2.997	172.03946[M + H-H_2_O]^+^, 162.05510[M + H-CO]^+^, 144.04449[M + H-H_2_O-CO]^+^, 116.04951[M + H-H_2_O-2CO]^+^	Urine (36–54, 3.524); bile (20–24, 0.426); stomach (6, 0.024)
C3	0.70	340.10287	C_15_H_18_O_8_N	0.521	322.09247[M + H-H_2_O]^+^, 304.08197[M + H-2H_2_O]^+^, 260.09192[M + H-2H_2_O-CO_2_]^+^, 206.08089[M + H-C_4_H_6_O_5_]^+^, 174.05484[M + H-C_5_H_10_O_6_]^+^, 164.07057174.05484[M + H-C_6_H_8_O_6_]^+^, 146.06000[M + H-C_6_H_8_O_6_-H_2_O]^+^, 136.07559[M + H-C_6_H_8_O_6_-CO]^+^, 122.05999[M + H-C_6_H_8_O_6_-C_2_H2O]^+^	Urine (24–36, 20.271); bile (20–24, 0.344)
C4	0.71	127.03858	C_6_H_7_O_3_	−3.075	83.04864[M + H-CO_2_]^+^	Urine (24–36, 2.557); liver (6, 0.0.154)
C5	0.79	162.05447	C_9_H_8_O_2_N	−2.993	144.04382[M + H-H_2_O]^+^, 134.05949[M + H-CO]^+^, 105.03305[M + H-C_2_H_3_ON]^+^	Urine (36–54, 101.501); feces (12–24, 8.460); bile (20–24, 0.277); stomach (6, 0.054); liver (12, 0.0.038)
C6	0.81	180.06503	C_9_H_10_O_3_N	−2.720	162.05437[M + H-H_2_O]^+^, 134.05952[M + H-H_2_O-CO]^+^, 105.03307[M + H-H_2_O-CO-CH_3_N]^+^	Urine (8–12, 109.725); bile (20–24, 2.565); liver (12, 0.076); stomach (3, 0.004)
C7	0.88	194.08064	C_10_H_12_O_3_N	−2.730	176.07001[M + H-H_2_O]^+^, 148.07516[M + H-H_2_O-CO]^+^, 120.08035[M + H-H_2_O-2CO]^+^, 91.05382[M + H-H_2_O-2CO-CH_3_N]^+^, 76.03896[M + H-C_8_H_6_O]^+^	Urine (36–54, 222.178); bile (20–24, 6.207); liver (12, 0.305); stomach (6, 0.091); feces (0–2, 0.049)
C8	0.92	164.07057	C_9_H_10_O_2_N	−0.214	146.05969[M + H-H_2_O]^+^, 136.07573[M + H-CO]^+^, 122.05989[M + H-C_2_H_2_O]^+^	Urine (24–36, 18.666); feces (2–4, 6.623); bile (16–20, 0.502); large intestine (1, 0.113); stomach (6, 0.102); liver (6, 0.049)
C9	1.10	167.07028	C_9_H_11_O_3_	0.055	149.05870[M + H-H_2_O]^+^, 121.06412[M + H-CO]^+^	Liver (0.083, 0.576); urine (0–2, 0.309); feces (4–6, 0.204)
C10	1.13	141.05414	C_7_H_9_O_3_	−3.408	105.03313[M + H-2H_2_O]^+^, 97.06436[M + H-CO_2_]^+^	Urine (8–12, 0.025)
C11	1.24	206.08061	C_11_H_12_O_3_N	−2.716	131.04919[M + H-C_2_H_5_O_2_N]^+^, 103.05421[M + H-C_2_H_5_O_2_N-CO]^+^	Urine (24–36, 9.382); bile (16–20, 3.133); feces (0–2, 0.116); stomach (0.33, 0.015)
C12	1.38	151.07503	C_9_H_11_O_2_	−2.159	133.06436[M + H-H_2_O]^+^, 123.07993[M + H-CO]^+^, 105.06962[M + H-H_2_O-CO]^+^	Urine (12–24, 0.416); feces (0–2, 0.058)
C13	1.63	193.04962	C_10_H_9_O_4_	0.439	123.04360[M + H-C_3_H_2_O_2_]^+^	Urine (24–36, 0.028)
C14	2.73	149.05949	C_9_H_9_O_2_	−1.450	131.04880[M + H-H_2_O]^+^, 103.05347[M + H-CO]^+^	Urine (24–36, 0.089); feces (24–36, 0.044)
C15	2.78	123.04377	C_7_H_7_O_2_	−2.324	105.03307[M + H-H_2_O]^+^, 95.04909[M + H-CO]^+^	Urine (36–54, 0.889); feces (24–36, 0.368); large intestine (2, 0.160); stomach (6, 0.091); liver (6, 0.044)

T_max_ is the time or time interval to reach in biological samples. R_max_ is the maximum peak area ratio of catabolic metabolites relative to the internal standard.

a Sorted by R_max_ from large to small.

### Excretion Study

**FIGURE 6:**
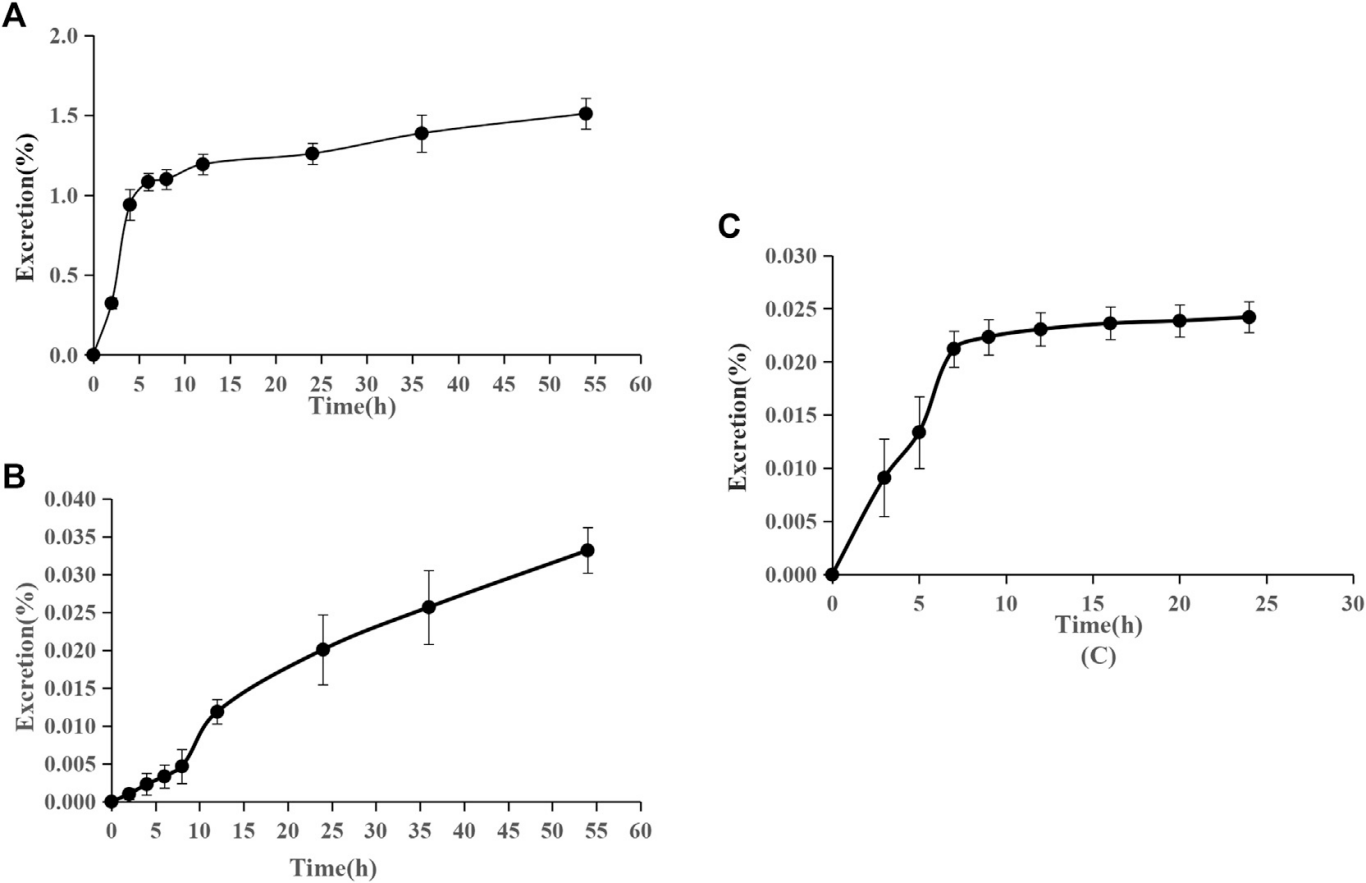
Accumulative excretion ratio of pinostrobin in urine **(A)**, feces **(B)**, and bile **(C)** after oral administration of 48.51 mg/kg pinostrobin (*n* = 5, mean ± SD).

The accumulative excretion ratio of pinostrobin is shown in Figure 6. The 54 h accumulative excretion ratios in urine and feces were 1.51% and 0.033%, respectively. The 24 h accumulative excretion ratio in bile was 0.024%. The excretion peak of pinostrobin in urine samples was observed 2–12 h after oral administration. After 24 h, a small amount of pinostrobin was detected in the urine. In feces, pinostrobin slowly reached its highest levels until 36 h and then gradually decreased. Similar to the urine excretion data, pinostrobin was rapidly excreted from the bile in the parent form from 3 to 9 h.

## Discussion

The pharmacokinetic results are consistent with those of previous studies (Hua et al., 2011; Sayre et al., 2013; Sayre et al., 2015). The significantly large V_z_ implied that pinostrobin preferentially binds to tissues and preferably remains within the body. Furthermore, pinostrobin showed double peaks in the concentration-time curve, similar to the pharmacokinetics studies of other flavonoid compounds that have been reported, which may be related to enterohepatic circulation or gastric emptying-regulated absorption (Xiong et al., 2015; Deng et al., 2017; Zeng et al., 2019). The accumulative excretion ratios in the urine remained below 1.51%, indicating that very little of the orally administered doses are excreted in the urine. A small amount of pinostrobin was detected in the urine after 24 h, which is consistent with previous reports using HPLC (Sayre et al., 2015). Pinostrobin slowly reached its highest levels in the feces until 36 h and was rapidly excreted from the bile from 3 to 9 h. Similar to urine excretion, the accumulative excretion ratios in the feces and bile are also very low. Therefore, it is likely that pinostrobin is mostly metabolized *in vivo* and plays a role in different organs.

Pinostrobin has been shown to have a significant protective effect against ethanol-induced gastric mucosal injury by scavenging free radicals produced by ethanol by activating cellular antioxidant defense. It can protect the gastric mucosa by reducing ulcer area and mucosal content and reducing or eliminating submucosal edema and leukocyte infiltration (Abdelwahab et al., 2011). Thus, the gastrointestinal tract (including the stomach, small intestine, and large intestine) is considered the primary target organ of pinostrobin in relieving gastrointestinal diseases. Pinostrobin accumulation was higher in the stomach, small intestine, and large intestine than in other tissues. Therefore, pinostrobin may effectively reduce peptic ulcers. In contrast, pinostrobin and its metabolites were poorly distributed in the tissues of the spleen, lung, heart, and kidney. Furthermore, very little pinostrobin was found in any examined tissues within 24 h, indicating no long-term accumulation in the tissues.

Owing to the high sensitivity and resolution of UPLC-LTQ orbitrap-MS/MS, more flavonoid metabolites and catabolic metabolites were detected in the biosamples. Pinostrobin underwent hydrogenation, hydroxylation, demethylation, decarbonylation, and acetylation to yield several other aglycones, including M5, M16, M11, M30, M23, and M7. Moreover, pinostrobin and the generated aglycones extensively undergo dehydrogenation, hydroxylation, and ketone conversion catalyzed by phase I metabolic enzymes and glucuronidation, sulfation, glycine conjugation, N-acetylcysteine conjugation, and taurine conjugation catalyzed by phase II metabolic enzymes in the gastrointestinal tract, liver, and other tissues. It is noteworthy that glucuronidation, decarbonylation, hydroxylation, demethylation, dehydrogenation, and glycine conjugation play essential roles in the metabolism of pinostrobin in rats.

## Conclusion

In this study, a series of rapid, reliable, and sensitive UPLC-LTQ orbitrap-MS/MS methods were established, validated, and applied to quantify pinostrobin in the plasma, urine, feces, bile, and various tissue samples collected from rats (Fu et al., 2018). After a single oral administration of pinostrobin, the pharmacokinetics showed a large apparent V_z_, indicating that pinostrobin preferentially binds to tissues to exert a therapeutic effect. Pinostrobin was mostly distributed in the gastrointestinal tract, suggesting that it may be an effective component of traditional Chinese medicines to treat peptic ulcers. The excretion study showed that the amount of pinostrobin excreted through the urine, feces, and bile in the parent form was less than 1.567%, indicating that it is mainly metabolized *in vivo*. Using UPLC-LTQ orbitrap-MS/MS, 30 flavonoid metabolites were identified or partially identified in biosamples collected after dosing. In addition, we proposed the metabolism pathways of pinostrobin in rats. This study systemically investigated the pharmacokinetics, tissue distribution, metabolism, and excretion of pinostrobin in rats. These results would be helpful for the interpretation of the pharmacokinetics and pharmacodynamics of pinostrobin as well as traditional Chinese medicines containing pinostrobin. However, further investigations are necessary to better understand the pharmacokinetics of pinostrobin in rats with peptic ulcer and the mechanism of its intervention on peptic ulcer.

## Data Availability Statement

The original contributions presented in the study are included in the article/**Supplementary Material**, further inquiries can be directed to the corresponding author.

## Ethics Statement

The animal study was reviewed and approved by the Experimental Animal Ethics Committee of Henan University of Chinese Medicine.

## Author Contributions

XS and SC conceived and designed the study. XS and XL analyzed the research data and wrote the manuscript. XS and XL performed the experiments.

## Funding

This work was supported by the National Natural Science Foundation of China (Grant No. 81773859), the National Science and Technology Major Project of the Ministry of Science and Technology of China (Grant No. 2012ZX09103201-024), and the Doctoral Research Fund Project of Henan University of Chinese Medicine (Grant No. BSJJ2018-07).

## Conflict of Interest

The authors declare that the research was conducted in the absence of any commercial or financial relationships that could be construed as a potential conflict of interest.
