# Supplementary material for: The Pharmacokinetics, Tissue Distribution, Metabolism, and Excretion of Pinostrobin in Rats: Ultra-High-Performance Liquid Chromatography Coupled With Linear Trap Quadrupole Orbitrap Mass Spectrometry Studies
Source: Front Pharmacol. 2020 Nov 26;11:574638. doi: 10.3389/fphar.2020.574638 (PMC7725875; doi:10.3389/fphar.2020.574638)
Supplement: Supplementary file 1 [file datasheet1.zip › Supplementary_Material/Supplementary Table S1.docx]

Table S1 Calibration curves, correlation coefficients and linear ranges of pinostrobin in different bio-samples

| **Samples** | **Calibration curve** | **Correlation coefficient(r)** | **Linear range(****ng/mL or ng/g)** |
| --- | --- | --- | --- |
|  |  |  |  |
| Plasma | Y=0.0172X+0.0955 | 0.9978 | 4-2000 |
| Heart | Y=0.0056X+4.4951 | 0.9961 | 8-4000 |
| Liver | Y=0.0062X+0.3373 | 0.9986 | 8-4000 |
| Spleen | Y=0.0037X+0.4207 | 0.9994 | 8-4000 |
| Lung | Y=0.0038X+0.2120 | 0.9997 | 8-4000 |
| Kidney | Y=0.0045X+0.3140 | 0.9972 | 8-4000 |
| Stomach | Y=0.0056X+0.4684 | 0.9976 | 8-10000 |
| Small intestine | Y=0.0053X+0.3639 | 0.9983 | 8-10000 |
| Large intestine | Y=0.0045X+0.3771 | 0.9983 | 8-10000 |
| Urine | Y=0.0040X+0.0515 | 0.9998 | 4-2000 |
| Faeces | Y=0.0073X+3.7316 | 0.9987 | 4-2000 |
| Bile | Y=0.0420X+0.3611 | 0.9979 | 4-2000 |
