# Supplementary material for: The Pharmacokinetics, Tissue Distribution, Metabolism, and Excretion of Pinostrobin in Rats: Ultra-High-Performance Liquid Chromatography Coupled With Linear Trap Quadrupole Orbitrap Mass Spectrometry Studies
Source: Front Pharmacol. 2020 Nov 26;11:574638. doi: 10.3389/fphar.2020.574638 (PMC7725875; doi:10.3389/fphar.2020.574638)
Supplement: Supplementary file 1 [file datasheet1.zip › Supplementary_Material/Supplementary Table S2.docx]

Table S2 Intra- and inter-assay precision, accuracy for the determining pinostrobin in rat plasma, urine and stomach homogenates (n=3 days, 6 replicates per day ).

| **Bio-sample** | **Nominol concentration**  **(ng/mL or ng/g)** | **Intra-day** | | **Inter-day** | |
| --- | --- | --- | --- | --- | --- |
|  |  | **Precision RSD(%)** | **Accuracy RE(%)** | **Precision RSD(%)** | **Accuracy RE(%)** |
| Plasma | 4 (LLOQ) | 8.7 | -10.6 | 9.4 | -5.0 |
|  | 10 | 7.3 | -13.2 | 10.7 | -8.3 |
|  | 400 | 3.7 | -3.8 | 6.3 | -3.0 |
|  | 2000 | 8.2 | -11.1 | 7.0 | -8.8 |
| Heart | 8 (LLOQ) | 12.6 | -13.0 | 18.2 | 7.6 |
|  | 20 | 11.0 | -12.9 | 12.9 | 9.8 |
|  | 800 | 9.5 | 5.1 | 9.2 | -6.4 |
|  | 4000 | 5.2 | -2.5 | 1.6 | 4.3 |
| Liver | 8 (LLOQ) | 9.3 | 6.7 | 15.2 | 8.3 |
|  | 20 | 6.9 | 9.0 | 14.5 | 5.8 |
|  | 800 | 8.9 | -8.7 | 11.1 | -3.4 |
|  | 4000 | 2.8 | 6.1 | 9.9 | 3.5 |
| Spleen | 8 (LLOQ) | 10.1 | 15.3 | 12.2 | 13.6 |
|  | 20 | 3.7 | 8.1 | 7.4 | 10.5 |
|  | 800 | 7.5 | -9.5 | 10.9 | -3.1 |
|  | 4000 | 6.6 | 2.0 | 3.2 | 5.4 |
| Lung | 8 (LLOQ) | 4.8 | -7.9 | 6.1 | -5.3 |
|  | 20 | 7.3 | -11.4 | 8.5 | -2.9 |
|  | 800 | 4.5 | 1.5 | 7.2 | 6.7 |
|  | 4000 | 3.4 | 2.3 | 3.1 | -1.3 |
| Kidney | 8 (LLOQ) | 9.1 | 13.2 | 14.0 | 10.2 |
|  | 20 | 7.6 | 10.4 | 4.3 | 6.4 |
|  | 800 | 4.6 | -7.3 | 6.6 | -8.1 |
|  | 4000 | 3.9 | -1.8 | 7.9 | 2.7 |
| Stomach | 8 (LLOQ) | 5.4 | -4.1 | 9.5 | -8.4 |
|  | 20 | 3.8 | -2.3 | 4.2 | -6.5 |
|  | 1000 | 2.6 | -1.7 | 5.7 | 8.5 |
|  | 10000 | 2.9 | -0.8 | 3.0 | -2.2 |
| Small intestine | 8 (LLOQ) | 15.1 | -10.7 | 18.0 | -9.9 |
|  | 20 | 4.9 | -3.2 | 6.7 | -5.2 |
|  | 1000 | 5.3 | 6.8 | 9.1 | -2.7 |
|  | 10000 | 7.1 | -5.7 | 7.3 | 1.2 |
| Large intestine | 8 (LLOQ) | 12.8 | 9.7 | 11.3 | 8.0 |
|  | 20 | 5.9 | 7.4 | 8.2 | 10.3 |
|  | 1000 | 4.8 | -4.3 | 9.6 | 5.2 |
|  | 10000 | 5.6 | 8.6 | 4.4 | 3.9 |
| Urine | 4 (LLOQ) | 8.8 | 14.7 | 6.2 | 11.5 |
|  | 10 | 2.2 | -5.4 | 1.9 | -7.4 |
|  | 400 | 3.2 | -8.4 | 5.3 | -5.5 |
|  | 2000 | 6.8 | 1.1 | 4.1 | -1.6 |
| Feces | 4 (LLOQ) | 11.7 | -9.4 | 12.7 | -9.2 |
|  | 10 | 6.3 | -12.4 | 7.0 | -6.1 |
|  | 400 | 8.4 | -2.6 | 5.5 | -1.9 |
|  | 2000 | 1.4 | 6.9 | 7.2 | 3.6 |
| Bile | 4 (LLOQ) | 7.8 | -9.1 | 15.7 | -5.6 |
|  | 10 | 10.6 | -4.2 | 9.7 | -5.1 |
|  | 400 | 4.7 | -1.2 | 5.9 | -3.5 |
|  | 2000 | 5.3 | -3.6 | 2.1 | -4.0 |
