# Supplementary material for: The Pharmacokinetics, Tissue Distribution, Metabolism, and Excretion of Pinostrobin in Rats: Ultra-High-Performance Liquid Chromatography Coupled With Linear Trap Quadrupole Orbitrap Mass Spectrometry Studies
Source: Front Pharmacol. 2020 Nov 26;11:574638. doi: 10.3389/fphar.2020.574638 (PMC7725875; doi:10.3389/fphar.2020.574638)
Supplement: Supplementary file 1 [file datasheet1.zip › Supplementary_Material/Supplementary Table S3.docx]

Table S3 Extraction recovery and matrix effect of pinostrobin and IS in rat plasma, urine and stomach homogenates (n=6).

| **Bio-sample** | **Nominal concentrations (ng/mL or ng/g)** | **Extraction recovery (%)** | **RSD(%)** | **Matrix effect (%)** | **RSD(%)** |
| --- | --- | --- | --- | --- | --- |
| Plasma | 10 | 83.1 | 12.9 | 91.4 | 10.9 |
|  | 400 | 90.2 | 7.6 | 81.5 | 3.5 |
|  | 2000 | 87.3 | 8.6 | 86.7 | 7.0 |
| Liver | 20 | 97.3 | 8.4 | 81.0 | 10.8 |
|  | 800 | 101.5 | 7.8 | 97.6 | 9.7 |
|  | 4000 | 105.6 | 3.1 | 102.0 | 6.3 |
| Heart | 20 | 81.8 | 11.8 | 89.7 | 11.1 |
|  | 800 | 94.9 | 14.7 | 96.4 | 9.6 |
|  | 4000 | 93.8 | 8.5 | 85.9 | 4.4 |
| Spleen | 20 | 98.7 | 4.5 | 95.2 | 6.6 |
|  | 800 | 103.9 | 5.0 | 106.6 | 7.7 |
|  | 4000 | 98.0 | 1.3 | 94.6 | 2.7 |
| Lung | 20 | 85.6 | 7.9 | 91.3 | 5.8 |
|  | 800 | 82.7 | 3.4 | 87.6 | 2.3 |
|  | 4000 | 88.2 | 5.6 | 84.7 | 3.2 |
| Kidney | 20 | 86.0 | 8.1 | 83.6 | 9.3 |
|  | 800 | 94.2 | 5.4 | 89.1 | 8.8 |
|  | 4000 | 90.5 | 6.5 | 87.4 | 3.6 |
| Stomach | 20 | 85.4 | 4.7 | 81.1 | 6.9 |
|  | 1000 | 93.6 | 3.8 | 93.4 | 3.3 |
|  | 10000 | 103.2 | 3.7 | 98.6 | 2.2 |
| Small intestine | 20 | 89.5 | 7.1 | 84.5 | 5.9 |
|  | 1000 | 88.6 | 4.6 | 90.4 | 7.3 |
|  | 10000 | 97.5 | 3.9 | 90.1 | 4.8 |
| Large intestine | 20 | 86.4 | 10.3 | 83.8 | 12.1 |
|  | 1000 | 85.5 | 5.7 | 91.6 | 8.2 |
|  | 10000 | 88.3 | 7.2 | 93.7 | 1.8 |
| Urine | 10 | 82.1 | 4.2 | 86.2 | 5.3 |
|  | 400 | 97.2 | 2.4 | 98.8 | 1.7 |
|  | 2000 | 101.8 | 1.9 | 99.5 | 2.1 |
| Feces | 10 | 85.3 | 9.4 | 102.1 | 11.6 |
|  | 400 | 90.6 | 6.2 | 92.7 | 9.5 |
|  | 2000 | 94.8 | 5.5 | 96.3 | 4.9 |
| Bile | 10 | 107.1 | 8 | 91.1 | 6.7 |
|  | 400 | 92.4 | 7.4 | 93.5 | 5.2 |
|  | 2000 | 104.6 | 6.1 | 90.7 | 2.8 |
