# Supplementary material for: The Pharmacokinetics, Tissue Distribution, Metabolism, and Excretion of Pinostrobin in Rats: Ultra-High-Performance Liquid Chromatography Coupled With Linear Trap Quadrupole Orbitrap Mass Spectrometry Studies
Source: Front Pharmacol. 2020 Nov 26;11:574638. doi: 10.3389/fphar.2020.574638 (PMC7725875; doi:10.3389/fphar.2020.574638)
Supplement: Supplementary file 1 [file datasheet1.zip › Supplementary_Material/Supplementary Table S4.docx]

Table S4 C_max_, T_max_ and AUC for pinostrobin in tissues after a single oral administration of 48.51 mg/kg pinostrobin to rats.

| **Tissues** | **Cmax (ng/g)** | **Tmax (h)** | **AUC (ng*h/g)** |
| --- | --- | --- | --- |
| Heart | 3072 | 3 | 20624 |
| Liver | 3592 | 0.75 | 5268 |
| Spleen | 1608 | 3 | 4602 |
| Lung | 2782 | 2 | 5468 |
| Kidney | 3378 | 0.75 | 4306 |
| Stomach | 7542 | 2 | 22454 |
| Small intestine | 7134 | 0.75 | 31272 |
| Large intestine | 3822 | 6 | 24586 |
