# Supplementary figures and images for: The Pharmacokinetics, Tissue Distribution, Metabolism, and Excretion of Pinostrobin in Rats: Ultra-High-Performance Liquid Chromatography Coupled With Linear Trap Quadrupole Orbitrap Mass Spectrometry Studies
Source: Front Pharmacol. 2020 Nov 26;11:574638. doi: 10.3389/fphar.2020.574638 (PMC7725875; doi:10.3389/fphar.2020.574638)

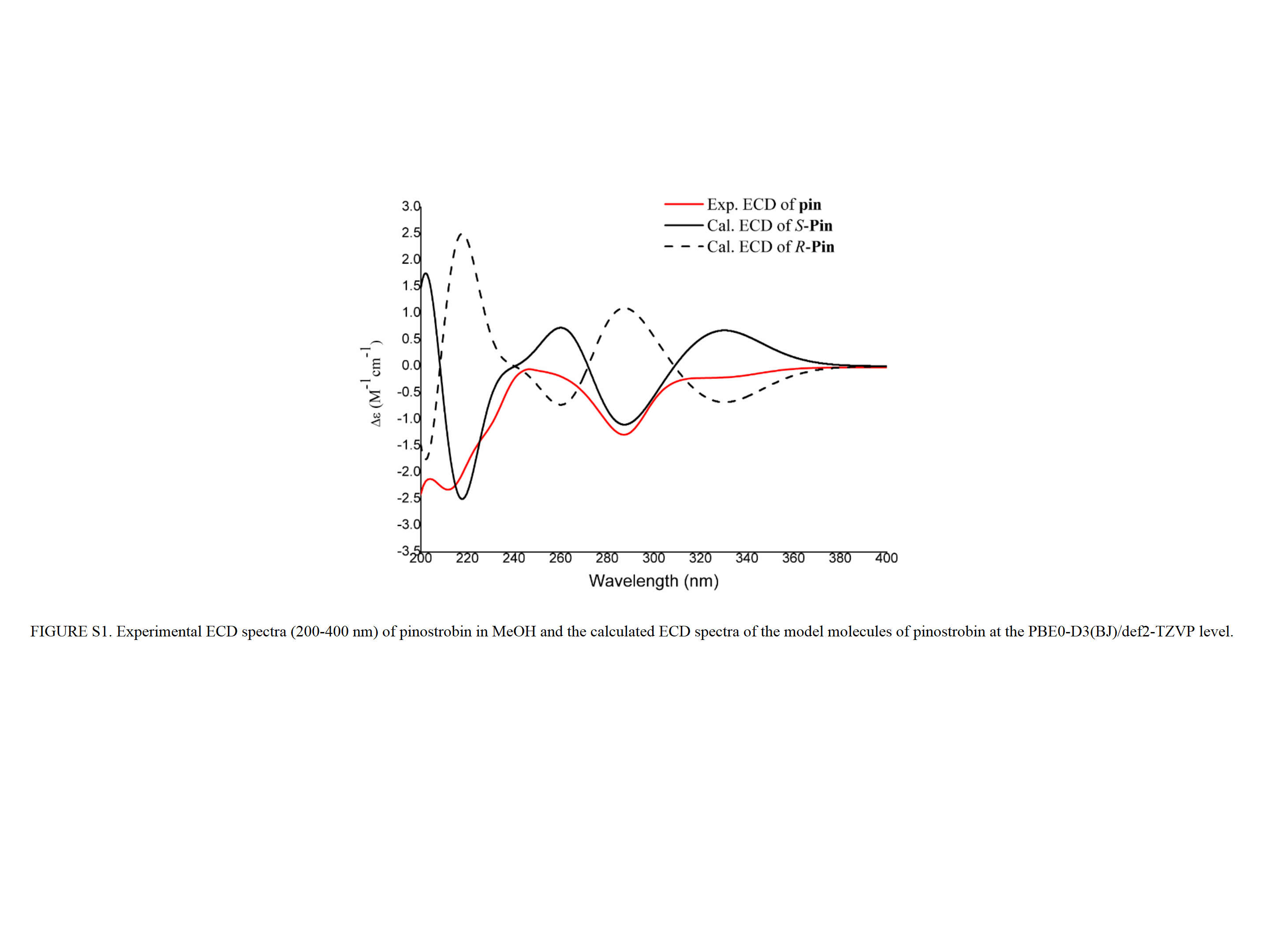

Supplement: Supplementary file 1 [file datasheet1.zip › Supplementary_Material/Supplementary Figure S1.tif]

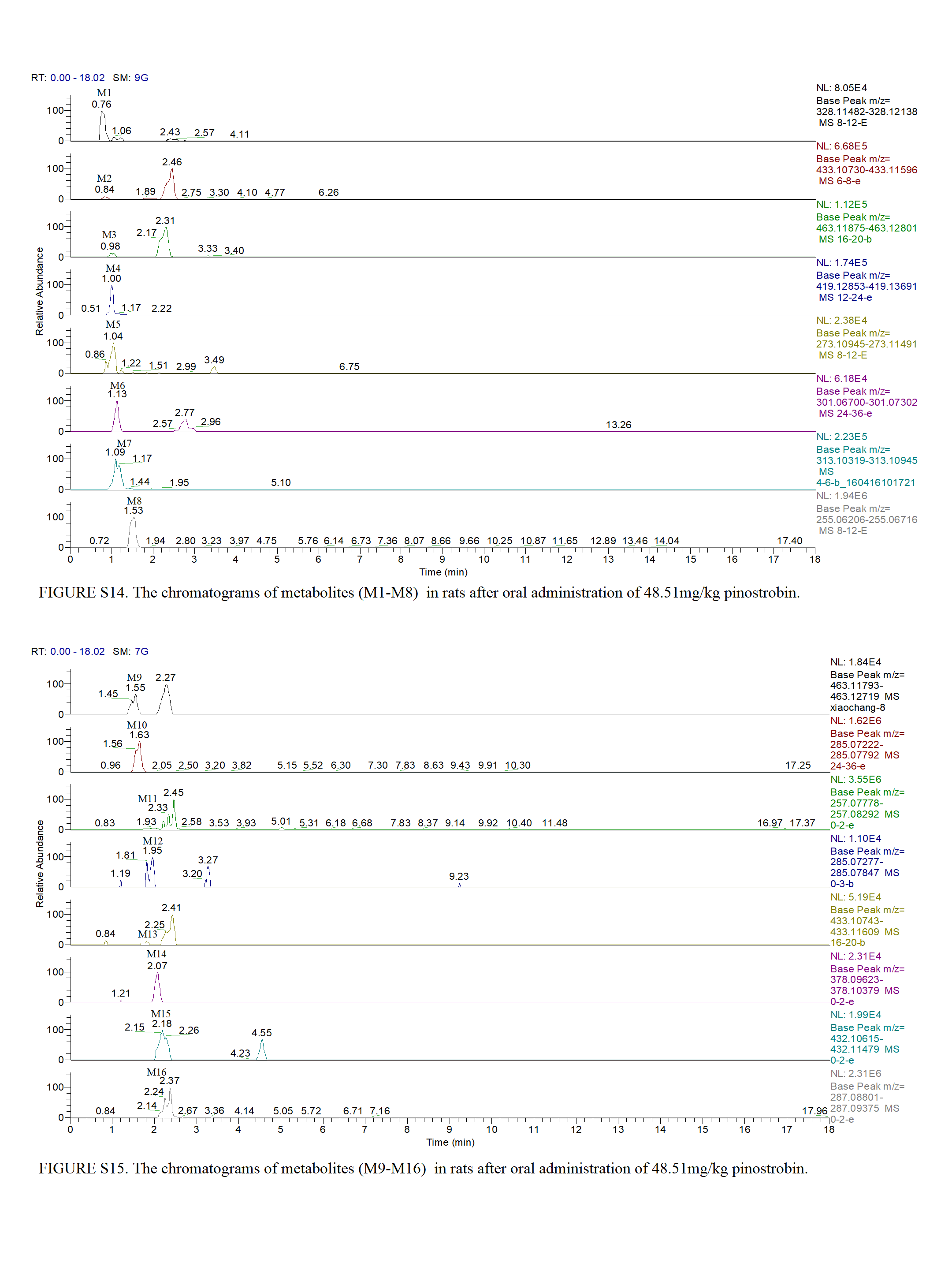

Supplement: Supplementary file 1 [file datasheet1.zip › Supplementary_Material/Supplementary Figure S14-S15.tif]

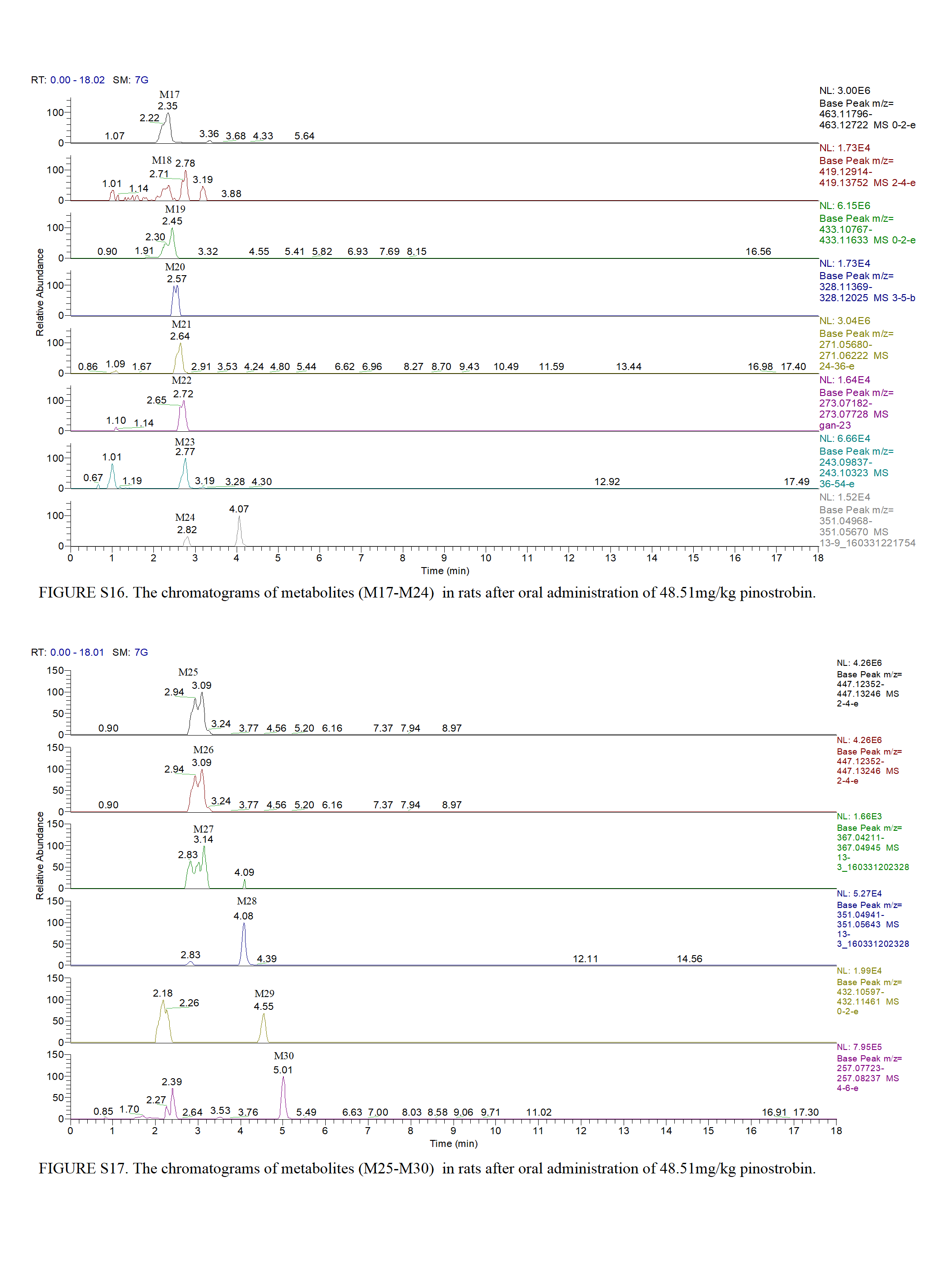

Supplement: Supplementary file 1 [file datasheet1.zip › Supplementary_Material/Supplementary Figure S16-S17.tif]

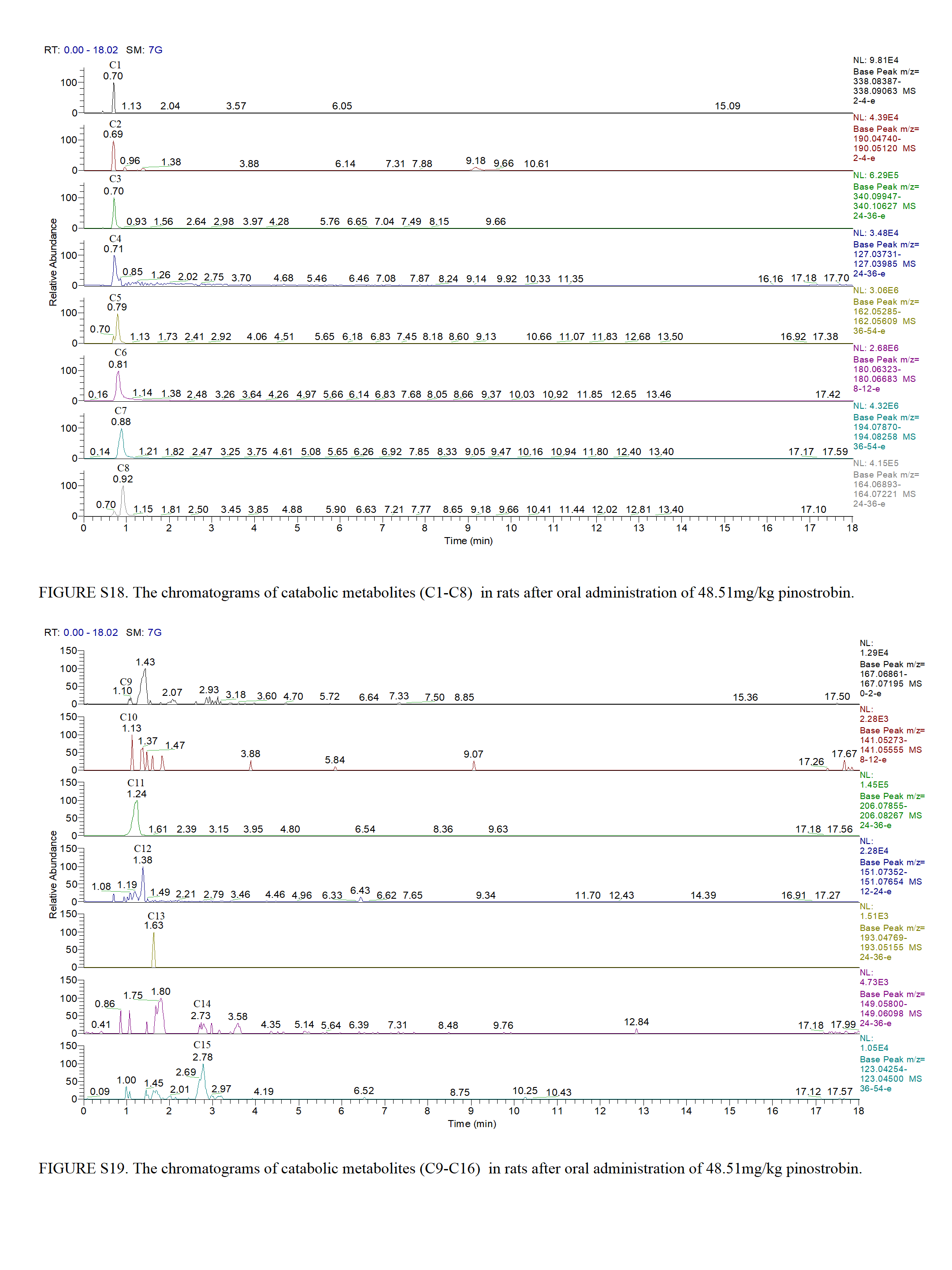

Supplement: Supplementary file 1 [file datasheet1.zip › Supplementary_Material/Supplementary Figure S18-S19.tif]

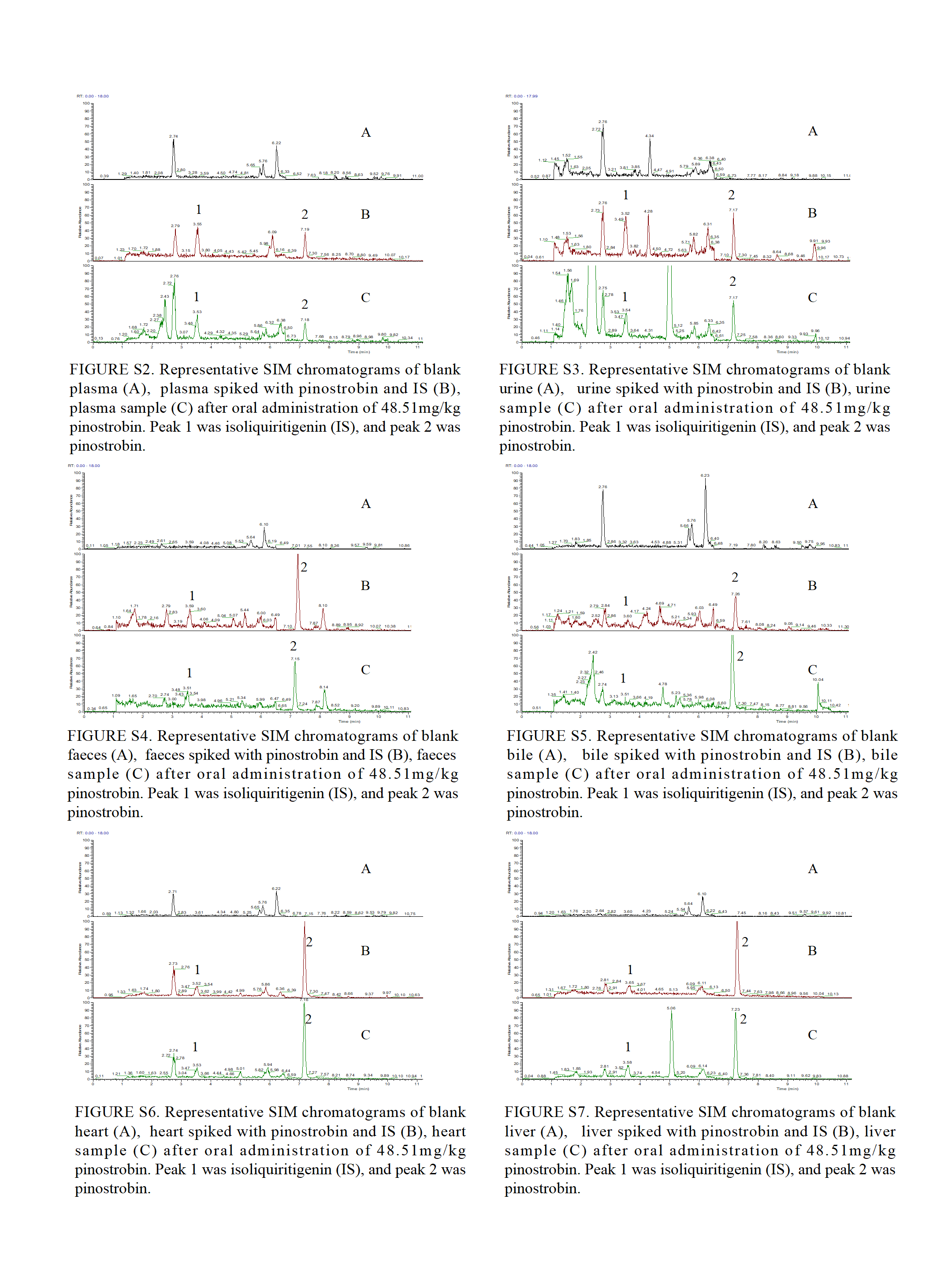

Supplement: Supplementary file 1 [file datasheet1.zip › Supplementary_Material/Supplementary Figure S2-S7.tif]

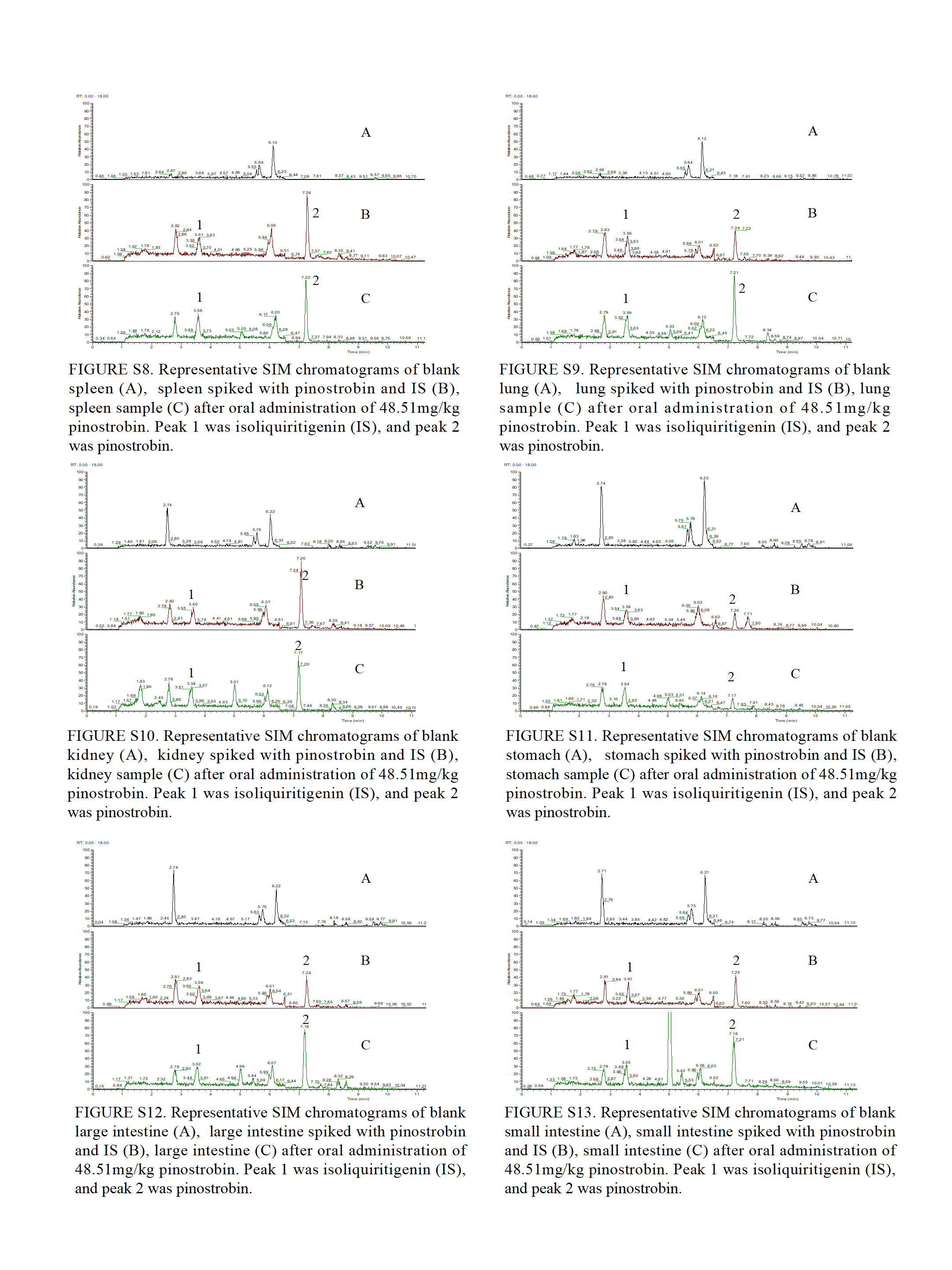

Supplement: Supplementary file 1 [file datasheet1.zip › Supplementary_Material/Supplementary Figure S8-S13.tif]
